# Composition of Sexual Fluids in *Cycas revoluta* Ovules During Pollination and Fertilization

**DOI:** 10.1007/s12229-021-09271-1

**Published:** 2022-01-01

**Authors:** Patrick von Aderkas, Stefan Little, Massimo Nepi, Massimo Guarnieri, Madeline Antony, Tokushiro Takaso

**Affiliations:** 1 Department of Biology, Centre for Forest Biology, University of Victoria, Victoria, BC V8W 3N5 Canada; 2 Department of Life Sciences, University of Siena, San Miniato, via Aldo Moro, 2, Via Pier Andrea Mattioli, 4, 53100 Siena, Italy; 3 Tropical Biosphere Research Center, University of the Ryukyus, 1 Senbaru, Nishihara-cho, Okinawa, 903-0213 Japan

**Keywords:** Amino acids, Carbohydrate, Cycad, Megagametophyte, Proteome, Sexual fluids

## Abstract

The composition of fluids that mediate fertilization in cycads is described for the first time. Using tandem mass spectrometry, proteomes of two stages of fluid production, megagametophyte fluid and archegonial chamber fluid production, are compared in *Cycas revoluta*. These were compared with the proteome of another sexual fluid produced by ovules, the pollination drop proteins. Cycad ovules produce complex liquids immediately prior fertilization. Compared with the pollination drops that mainly had few proteins in classes involved in defense and carbohydrate modification, megagametophyte fluid and archegonial chamber fluid had larger proteomes with many more protein classes, e.g. proteins involved in programmed cell death. Using high-performance liquid chromatography, megagametophyte fluid and archegonial chamber fluid were shown to have elevated concentrations of smaller molecular weight molecules including glucose, pectin and glutamic acid. Compared to megagametophyte fluid, archegonial chamber fluid had elevated pH as well as higher osmolality.

**Supplementary Information:**

The online version contains supplementary material available at 10.1007/s12229-021-09271-1.

## Introduction

**Fig. 1:**
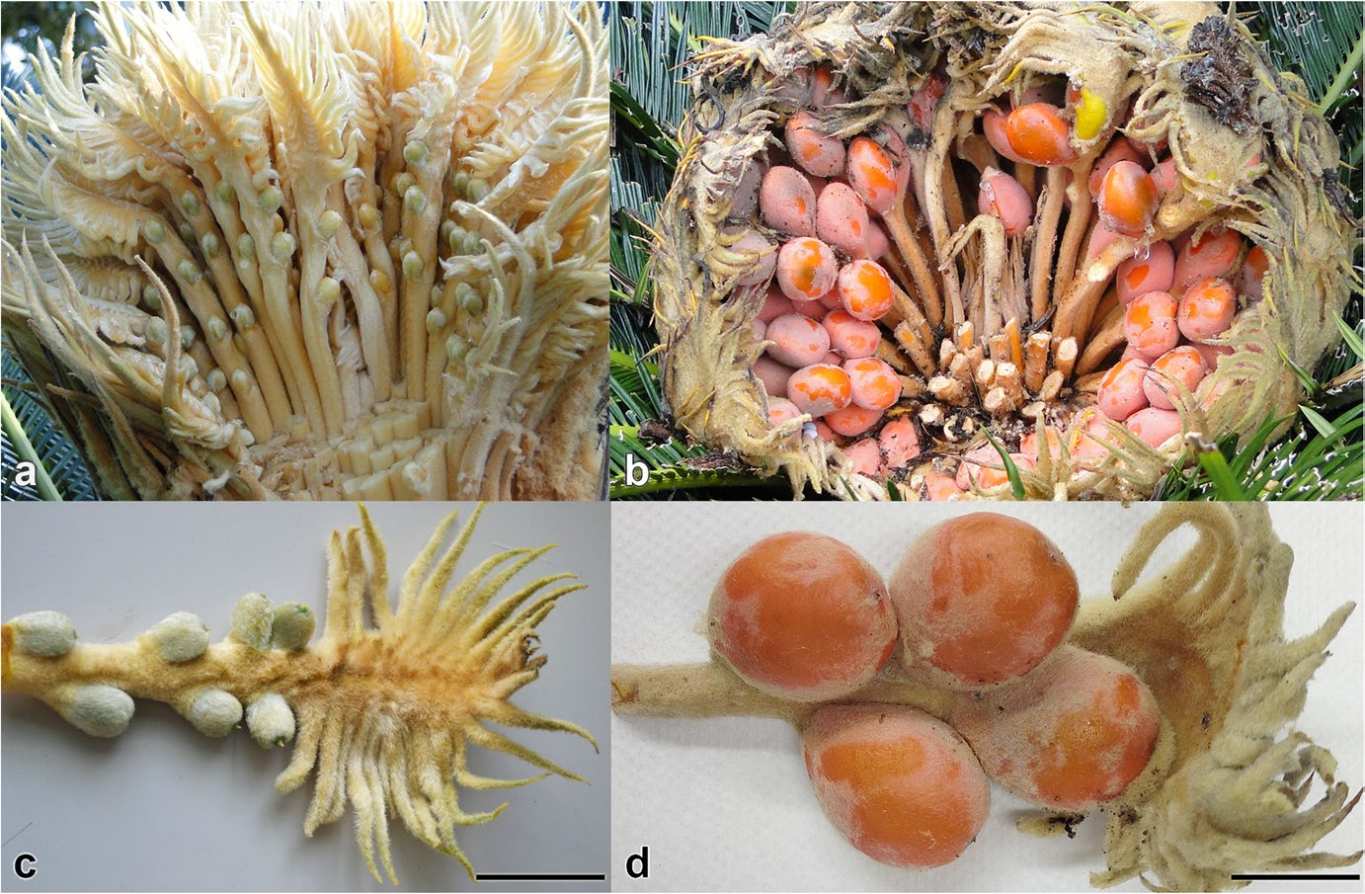
Megasporophylls and ovules of *Cycas revoluta* at the two developmental stages sampled for sexual fluids. **a** Megasporophylls exposed in the field during pollination drop secretion from ovules, which are light green in colour **b** Megasporophylls exposed in the field around time of fertilization inside orange-red colored ovules. **c** An isolated megasporophyll bearing ovules at the time of pollination drop secretion. **d** An isolated megasporophyll bearing receptive ovules, i.e. at the time of fertilization

**Fig. 2:**
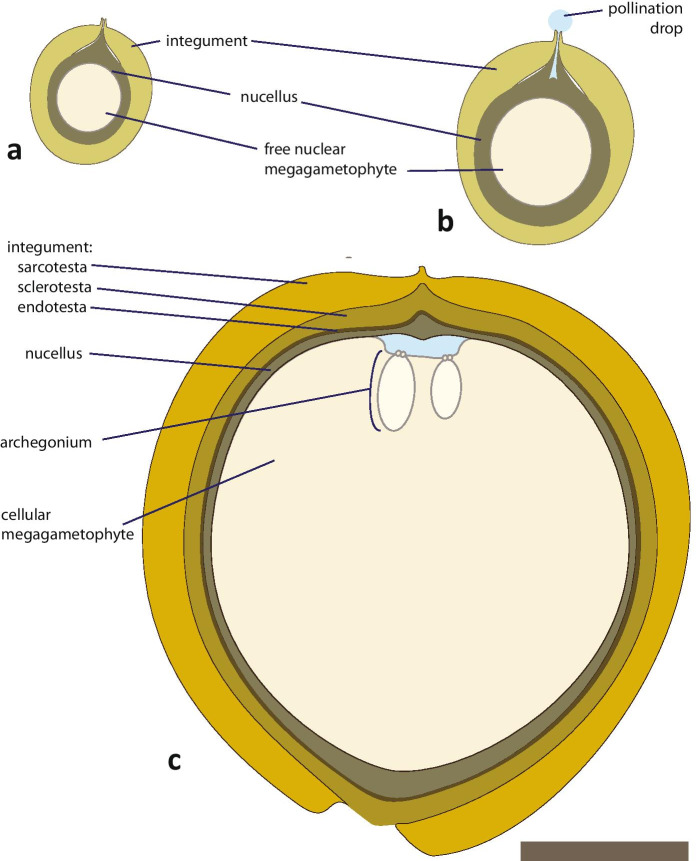
Schematic of ovule developmental stages around time of fluid collections; all ovules are to scale. **a** Immature ovule just prior to pollination drop production with nucellar apex protruding into the micropyle. **b** Ovule with a pollination drop coinciding with cellular breakdown of the nucellar apex to form a pollen chamber. **c** Ovule around time of fertilization with differentiated layers of integument containing a cellularized megagametophye bearing two archegonia at its apex at the base of the archegonial chamber. The nucellus, which caps the megagametophyte bears maturing microgametophytes which will release swimming sperm at the time of fertilization. Bar = 1 cm

In gymnosperms, pollination and fertilization occur in distinctly separated stages that are mediated by fluids. During pollination, pollen is captured by young ovules; various pollen capture mechanisms occur that are assemblages of physiological and morphological adaptations (Williams, 2009). A key component is an ovular fluid secretion known as a pollination drop that coordinates the formative male–female interactions. It draws pollen into the ovule’s interior where it germinates (Jin et al., 2012). Because pollination drops also contain antimicrobial enzymes, they have a defensive function (Coulter et al., 2012; Pirone-Davis et al., 2016). Pollination drops can be even more complex: in insect-pollinated gymnosperms, pollination drops have elevated carbohydrate concentrations that serve as a reward (Nepi et al., 2017). Pollination drops are widely distributed among gymnosperms (Little et al., 2014), but homologous ovular secretions are relatively uncommon among angiosperms (Willemse et al., 1995). Pollination drops are produced within the young ovule by the by the nucellus. Following pollination and pollen germination, the pollen tube grows. In many gymnosperms, this is a long process, taking weeks, if not months before the microgametophyte differentiates and delivers gametes (Williams, 2009). At the same time, the ovule grows and differentiates a megagametophyte bearing archegonia that contain receptive eggs. Fertilization can occur in one of two ways in gymnosperms: siphonogamy or zoidogamy. Siphonogamy is characteristic of more advanced clades. A pollen tube penetrates between the neck cells of the archegonium and releases its two sperm directly into the egg (Bruns & Owens, 2000). In zoidogamy, characteristic of more anciently derived clades, such as *Ginkgo* and cycads, a pollen tube releases two gametes into a fluid located within the ovule in a region between nucellus and megagametophyte (Figs. 1, 2). The flagellated gametes (spermatozoids) must swim under their own power to the archegonia and then penetrate the archegonium to reach and then fertilize the egg (Ikeno, 1896; Wang et al., 2014). The difference between zoidogamy and siphonogamy is, therefore, not only the difference between indirect and direct delivery of gametes, but between the production of a fluid for fertilization and the absence of such a fluid, respectively. Fluid production during fertilization of zoidogamous species is poorly understood and fluid composition is completely unknown. This is in sharp contrast to pollination drops, the composition of which is relatively well understood (Little et al., 2014).

Gymnosperm sexual fluids are low in volume, ephemeral, and often difficult to access, making their study challenging. For example, even if pollination drops are readily accessible at surfaces of exposed ovular surfaces, their low volumes would require a great deal of effort to accumulate sufficient volumes for analysis. Collection is further hampered in those species with unpredictable phenological patterns of secretion (O’Leary & von Aderkas, 2006). Like pollination drops, fluids involved in fertilization are short-lived, present for only a few days in cycads (Takaso et al., 2013) and *Ginkgo* (Friedman, 1987). In contrast to pollination drops, fertilization fluids are produced deep within the ovule, adding further complications for sampling. Beyond these difficulties posed for collection, such short timeframes underscore how tightly events are coordinated.

Stringent coordination of sperm release with female receptivity to achieve fertilization is found not only in zoidogamous seed plants, but in the free-sporing, early clades of land plants, such as mosses and ferns. For example, immediately before fertilization, mosses release the contents of archegonial cells, e.g. neck canal cells and ventral canal cells. Material released by their archegonia has a chemoattractant that draws spermatozoids to the egg (Ziegler et al., 1988). Recently, moss sperm were found to have an ionotropic glutamate receptor; the ligand remains unknown (Ortiz-Ramirez et al., 2017). Homologous proteins involved in sperm chemotaxis occur in many organisms including mice and humans. It is not just that liquid is required for sperm to swim, but the liquid is supplemented with substances to make fertilization more efficient. In cycads, the fluid is produced by cells at the apex of the megagametophyte (MF-megagametophyte fluid). Production is copious and the fluid subsequently flows into the adjacent archegonial chamber (Figs. 2, 4; Takaso et al., 2013). Similar to mosses and ferns, archegonia of cycads and *Ginkgo* also release compounds. In cycads, this emission mixes with MF already in the archegonial chamber: sperm swim in this admixture we here call archegonial chamber fluid (ACF).

**Fig. 3:**
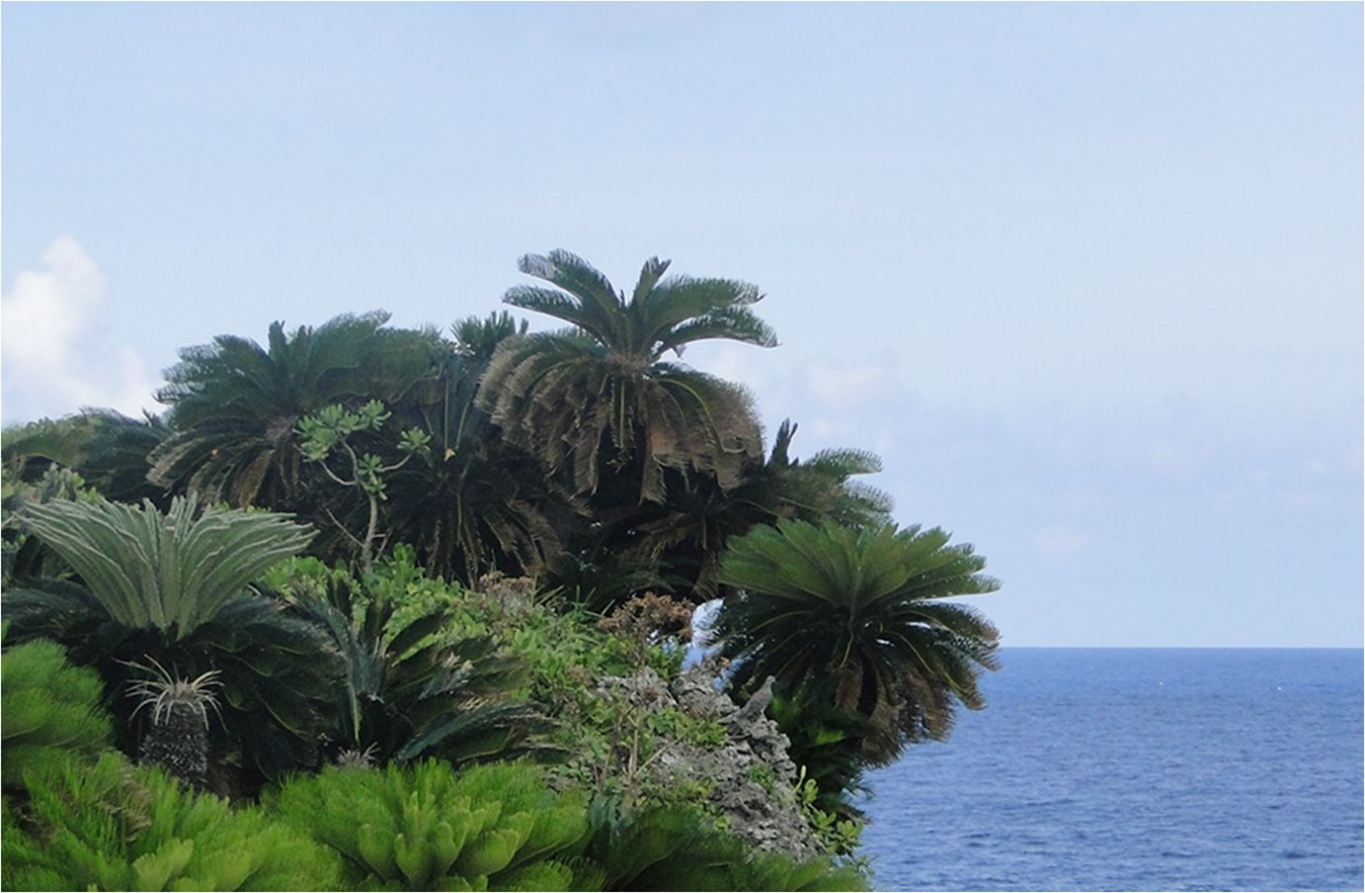
One of the collection sites on Iriomote Island, Okinawa, showing a native stand of *Cycas revoluta* from which several plants were sampled for this study

This paper focuses on the question of whether MF and ACF differ in their compositional profiles and functional roles. Further, we wish to know how these megagametophtye-derived fluids differ from the nucellus-derived pollination drop. We collected sexual fluids of *Cycas revoluta* (sago palm) within its native range in southern Japan (Fig. 3; Kyoda & Setoguchi, 2010). We analyzed proteomic profiles of MF, ACF, and pollination drops. Additionally, we measured osmotic concentration, pH, and the composition of both amino acids and carbohydrates. *Cycas* is rapidly diversifying genus (Mangka et al., 2020) within the Cycadophyta (Stevenson, 1992), an ancient group of seed plants that bears similarities with even more ancient seed ferns (von Aderkas et al., 2018). Regulation of microgametophytes by megagametophyte-derived sexual fluid is considered to be a plesiomorphic suite of characteristics in the evolution of seed plants (Takaso et al., 2013; von Aderkas et al., 2018).

## Materials and Methods

### Plant material

Ovules from both cultivated and wild plants of *Cycas revoluta* Thunb. were used. All plants are located in north-western Iriomote Island, Okinawa Prefecture, Japan. Locations of 24 female plants from which samples were collected are given in Supporting Information Table S1.

### Collection of Megagametophyte Fluids (MF) and Archegonial Chamber Fluids (ACF)

**Fig. 4:**
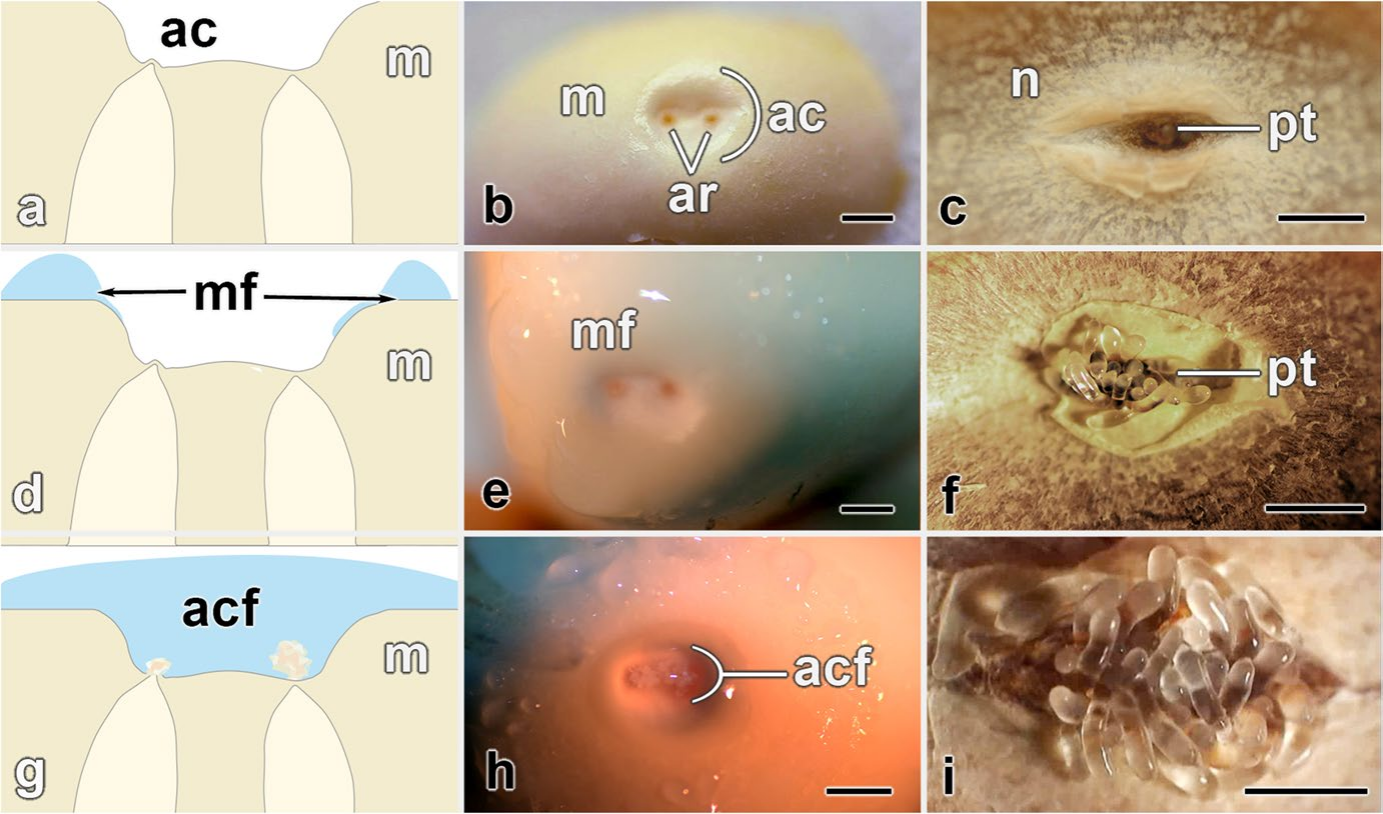
Genesis of prefertilization sexual fluids from megagametophytes of *Cycas revoluta* ovules. **a** The apex of the megagametophyte (m) had a depression at its apex, the archegonial chamber (ac), at the bottom of which two archegonia were found (in white). **b** In the days before fluid production, the apex of the megagametophyte was dry and the archegonial chamber (ac) empty. Two archegonia (ar) can be seen. **c** The roof of the archegonial chamber is the underside of the nucellus, which, at this stage, typically has a fissure into which the pollen was expanding. At this time, pollen tubes (pt) were either not visible, or just becoming visible. **d** The fluid formed on the megagametophyte surface, but not in the archegonial chamber. This was collected and is referred to as megagametophtye fluid (MF). **e** Fluid was present on the megagametophyte apex, but not in the archegonial chamber. **f** At this stage the pollen tubes were visible as they expanded from the nucellus. **g** The fluid has flowed into the archegonial chamber and soon after, the archegonia ejected flocculent materials to form the archegonial chamber fluid (ACF). **h** This flocculent material accumulated on the floor of the archegonial chamber in the vicinity of the archegonia from which it had been released. **i** The pollen expanded further into the chamber and was ready to release sperm. At the tips of the pollen tubes, white regions were visible in which the two sperm cells were maturing. All bars = 1 mm

Six plants were selected for manual pollination in mid-to-late May. Ovules were subsequently collected just before fertilization in August 2018. All ovules were removed from plants that had a promising number of ovules in which pollen tubes were seen to extend from the nucellus into the archegonial chamber (Fig. 4). Ovules were then cut into two halves. The chalazal half was discarded. The remaining half, the micropylar portion, was further dissected so that the lateral edges allowed the integument and apically free nucleus to be removed cleanly, leaving an exposed megagametophyte apex.

The following steps were carried out using nitrile gloves and clean equipment to avoid contamination. The megagametophtye apices were placed in glass Petri dishes lined with Whatman No. 1 filter paper that had been wetted with filtered water. Megagametophyte halves were left for approximately a day before megagametophyte fluid (MF) was collected from the upper megagametophyte surface. Archegonial chamber fluid (ACF), which is MF plus the materials emitted from the archegonium, were collected separately with glass micropipettes, immediately frozen and stored.

### Pollination Drops

Pollination drops (Fig. 2) were collected from ten plants over a 3-week period from mid- to late May. Receptive megasporophylls were removed from each plant and their cut ends placed in water. The megasporophylls were kept inside chambers to maintain a high relative humidity. Pollination drops were collected daily with 50 µL glass micropipettes as previously described for other pollination drops (Prior et al., 2018). To have an amount sufficient for mass spectrometric analysis, pollination drops were pooled.

### Sample Analysis

#### Electron Microscopy

Dissected samples including both megagametophtye and nucellus were fixed in 2% glutaraldehyde in 0.10 M cacodylate buffer, pH 7.0. After buffer washes, the samples were dehydrated in 50% ethanol for 10 min and then blockstained in 5% uranyl acetate in 50% ethanol for 60 min. Dehydration was continued using a graded ethanol series for 10 min per step in 70, 80, 90, 95, and 100%. The 100% ethanol step was repeated twice more before the samples were infiltrated through a graded series of ethanol and Spurr’s resin and embedded into pure Spurr’s resin and cured. Polymerized samples were sectioned on a Reichert Ultracut E ultramicrotome.

Sections were stained with uranyl acetate and Reynold’s lead citrate and viewed in a JEOL JEM-1011 transmission electron microscope. Images were captured using a Gatan Erlangshen ES1000W CCD camera.

#### Proteomics of MF and ACF

Protein content of MF and ACF of four individuals were measured using a standard Bradford test (Bradford, 1976).

Samples of raw unprocessed fluids (20 µL each) from various individuals were mixed with 5 µL NuPage Mes SDS buffer and 1 µL of 1 M dithiothreitol (DTT) and boiled for 10 min. Samples were loaded on to a NuPage Novex 4 gel (12% Bis–TRIS precast gel) along with 5 µL of BLUelf Prestained molecular ladder (FroggaBio-Bio-Helix Co. Ltd.). The gel was run for 25 min at 200 V, then fixed with 40% ethanol:10% acetic acid solution for 10 min, then stained in 0.1% Coomassie Brilliant Blue. The following day, it was de-stained for 5 h before photographing.

To establish the types of proteins in the samples, an analysis was carried out on protein bands cored from a sodium dodecyl sulfate–polyacrylamide gel electrophoresis (SDS-PAGE) gel. Two lanes from a gel in which megagametophyte and archegonial fluids from one cycad were run (as described above) and were subsequently cut into 16 equal pieces, then processed and analyzed as follows: samples were reduced with DTT for 30 min at 37 °C. Cysteine bonds were alkylated for 30 min at 37 °C with iodoacetamide. Following 16 h of porcine trypsin (2 µg Promega) digestion at 37 °C the samples were desalted by passing through a Waters HLB Oasis column. They were concentrated by SpeedVac and stored at − 80 °C until analysis.

Peptide mixtures were rehydrated and separated according to previously published methods (Prior et al. 2013). Samples were introduced into an LTQ Orbitrap Velos mass spectrometer equipped with a Nanospray II source (Thermo Fisher Scientific). Solvents were A: 2% acetonitrile, 0.1% formic acid; B: 90% acetonitrile, 0.1% formic acid. Samples were separated by a 90 min gradient (0 min: 5% B; 80 min: 45% B; 2 min: 90% B; 8 min: 90% B).

Raw liquid chromatograph-tandem mass spectroscopy (LC–MS/MS) files were converted to Mascot files (MGF) using Proteome Discoverer 1.4, Thermo Fisher Scientific (www.thermofisher.com). Mascot files were processed with PEAKS 6 (Bioinformatics Software Inc., Waterloo ON, Canada) with Peaks DB and Spider searches enabled against the SWISS-PROT database, the sequences downloaded from UniProtKB, and searches were performed using the viridiplantae organism group. The settings used were as follows: instrument type set as Fourier transform ion cyclotron resonance (FT-ICR)/Orbitrap; high energy Collision Induced Dissociation (CID) as fragmentation mode; parent ion error tolerance 8 ppm; fragment ion error tolerance 0.60 Da; precursor mass search type: monoisotopic; trypsin as proteolytic enzyme; up to one missed cleavage allowed; carbamidomethylation as a fixed modification; deamidation and oxidation as variable modifications; max variable PTM per peptide: 3. Those proteins with gene ontogeny annotations were then processed through Scaffold Software (see Supporting Information Methods S1).

#### Label-free Quantitation Mass Spectrometry of MF and ACF

A label-free quantitation experiment was done using MF and ACF from four individuals. As a result of establishing the protein concentrations by Bradford assay of each sample, we were able to work with an equal mass of protein per sample. Processing and analysis is described in detail as follows. Samples were processed by liquid chromatography and injected into an LTQ Orbitrap Velos. RawMeat was used to process the base peak chromatograms, and MaxQuant was used to process the raw files using default parameter settings. Andromeda software was used to search gymnosperm data within UniProt-SwissProt. The basis of the label-free quantitation is the MaxLFQ algorithm. The results were processed using the Perseus software platform (see Supporting Information Methods S1).

#### Proteomics of Pollination Drops

Processing of pollination drops by liquid–liquid separation and subsequent mass spectrometric analysis and data processing followed previously published methods (Prior et al., 2013, 2018). Detailed description is provided in Supporting Information Methods S1.

#### Sugar Analysis of MF and ACF

MF and ACF from four individuals were analyzed. These were run identically to previously published sugars analyzed in pollination drops (Nepi et al., 2017). Samples were analyzed for sugar content using isocratic HPLC (see Supporting Information Methods S1). In addition, the concentration of pectins was measured. It was expressed as galacturonic acid equivalents (mg ml^−1^). Because the pollination drop sample was of insufficient volume to be able to perform all types of analysis undertaken in this study, we did not measure carbohydrates in the pollination drop.

#### Amino Acid Analysis of MF and ACF

MF and ACF from ovules from the same four individuals above were analyzed. Amino acid analysis was performed by gradient HPLC with an AccQtag system column as previously published (Nepi et al., 2017). Details are in Supporting Information Methods S1. Because the pollination drop sample was of insufficient volume to be able to perform all types of analysis undertaken in this study, we did not measure amino acids in the pollination drop.

#### pH of MF and ACF

Eight MF and four ACF samples were thawed and measured at room temperature using a calibrated Thermo Scientific Orion Micro pH Electrode connected to an Accumet 900 pH meter (Fisher Scientific).

#### Osmotic Concentration of MF and ACF

Freezing-point depression was used to determine osmotic concentration. The samples were analyzed using a phase-contrast microscope (Olympus BHT) equipped with a freezing stage controlled by a Nanolitre Osmometer (Clifton Technical Physics, Hartford, NY). Samples were loaded into the sample holder with 25 µL Pressure-Lok Precision Analytical Syringe (VICI Precision Sampling). Samples were flash-frozen to − 40 °C and then slowly thawed until the smallest ice crystal was in equilibrium, at which time the osmolarity was recorded. Calibration was done using standard solutions of NaCl, i.e. 0 and 3000 milliOsmole l^−1^ (Osmol). Between readings, the sample holder was thoroughly cleaned by sanitation in a detergent solution that was subsequently washed off, followed by a rinse of 70% ethanol rinse and air drying. The loading syringe was cleaned after each sample by repeated sequential rinsing with double-distilled deionized water (ddH_2_0), detergent solution, ddH_2_0, and 70% ethanol.

## Results

### Stages in Megagametophyte Fluid Production

Ovules were sampled during a period from before to after fertilization. Based on inspection of nucellus and megagametophyte apex, these were divided into three stages (Fig. 4). The first stage was immediately before secretion: these megagametophytes had dry upper surfaces (Fig. 4a, b) and pollen tubes were not yet protruding from the nucleus, i.e. they could not yet be seen (Fig. 4c). As there were no fluids to collect from these they served only as a guide, i.e. pre-secretion samples. Isolated megagametophytes of such apices never produced fluids. The second stage of megagametophyte had upper surfaces that were initially dry, but within a day of being isolated, these same apices readily produced megametophyte fluid (MF) from the surface surrounding the archegonial chamber (Fig. 4d, e). At the time of isolation, these ovules could be distinguished from those of the previous stage by the emergence of pollen tubes from the nucellus (Fig. 4f). Samples isolated at the third stage produced megagametophtye fluids that flowed into the archegonial chamber (Fig. 4g, h). A typical ovule at this stage had a nucleus from which pollen protruded prominently (Fig. 4i). MF triggered separation of neck cells of the archegonia. This was followed almost immediately by expulsion of archegonial contents. These were of a flocculent nature. These expelled cellular fluids remained on the floor of the archegonial chamber (Fig. 4g, h). The combination of this ejected material with MF makes up what we are calling the archegonial chamber fluid (ACF). At the time of these last collections, pollen tubes had expanded (Fig. 4i). Once wetted by submersion in the fluids, they burst, releasing their gametes. Fertilization followed. Soon after fertilization, all megagametophyte surfaces became dry (not shown).

The sources of the fluids differed in that MF was produced by hundreds if not thousands of cells over a wide area of the apex of the megagametophyte, whereas archegonial secretions were point emissions from one or two archegonial cells. There was visual evidence of differing sol–gel properties of the two types of fluids: The colloidal, flocculent nature of the archegonial emission was different than the clear liquid of MF.

**Fig. 5:**
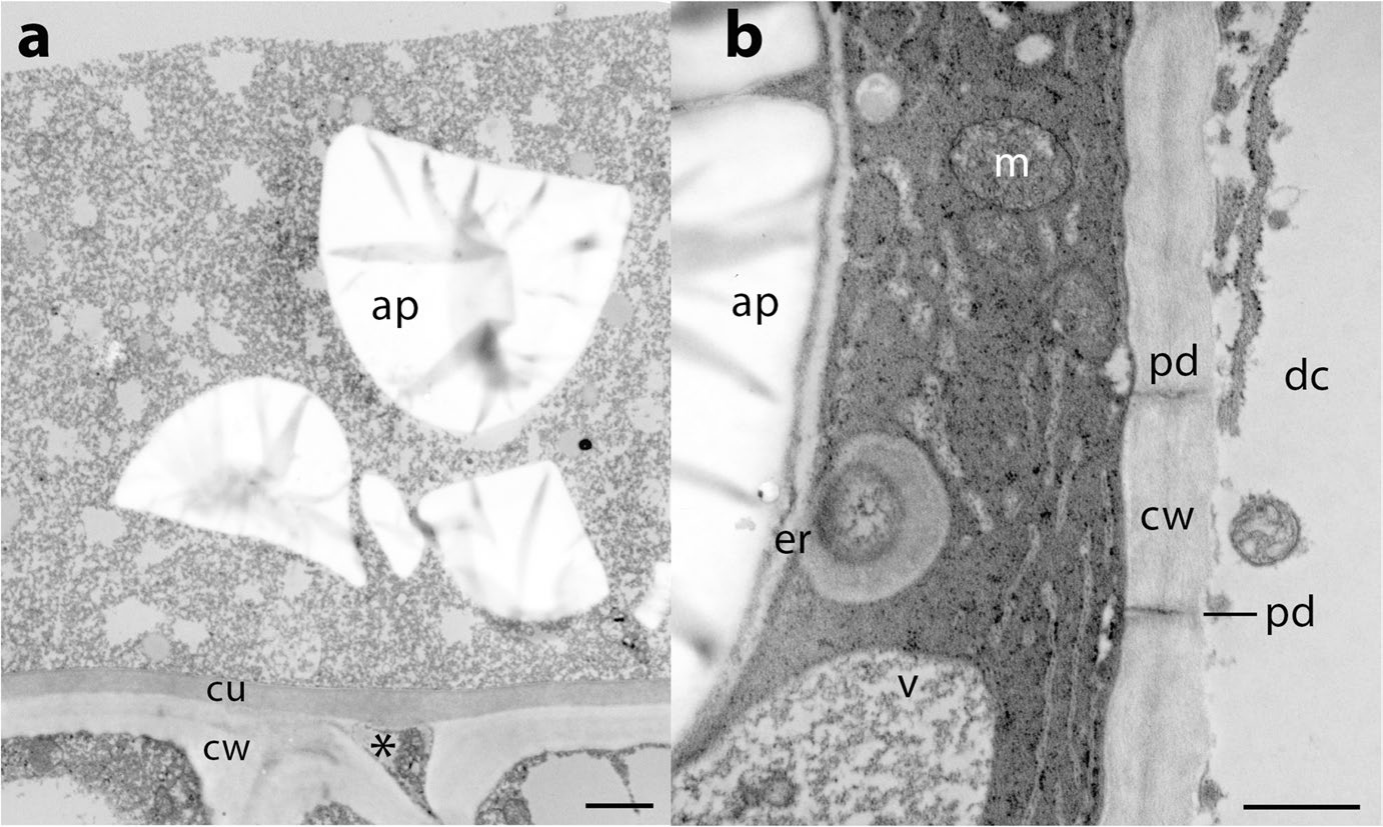
Transmission electron micrographs of epidermis of megagametophyte apex of *Cycas revoluta*. **a** Epidermal cells are under a thick layer of debris, including amyloplasts (ap) debris in the apoplast. The epidermal cells have a cuticle (cu) above the cell wall (cw). Portions of released cytoplasm can be seen in the intercellular space (asterisk) below the cuticle. Bar = 2 µm. **b** In a cell on the left that is undergoing PCD, endoplasmic reticulum (er) surround the amyloplast and engulf cytoplasm. Mitochondria (m) have poor membrane structure, and vacuoles (v) contain flocculent material. A cell wall (cw) with two plasmodesmata (pd) is visible. In the adjacent dead cell (dc) to the right, plasma membrane is absent and all that remains of the cytoplasm are traces of organelles. Bar = 0.5 µm

MF constituted the bulk of the fluid volume, which had a substantial solute component. A mass of accumulated debris could be seen on the surfaces of samples processed for transmission electron microscopy. Debris including starch grains were found in the apoplastic regions of the ovule (Fig. 5a). The materials originated from cells of the epidermal layer of the megagametophtye apex, many of which were undergoing cell death (Fig. 5b). These dying cells showed many unusual organellar features: endoplasmic reticulum is seen engulfing material in the vicinity of the amyloplast. Mitochondrial structure is diminished. Some cells have largely been emptied of their contents.

### Proteins in Sexual Fluids

**Fig. 6:**
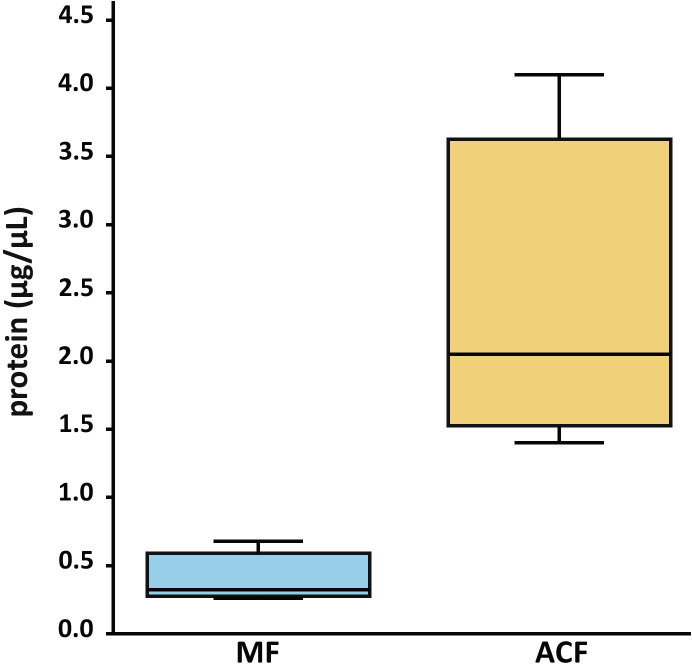
Boxplot of protein concentrations (µg/µL) of megagametophyte fluid (MF, blue) and archegonial chamber fluid (ACF, yellow) of *Cycas revoluta*

**Fig. 7:**
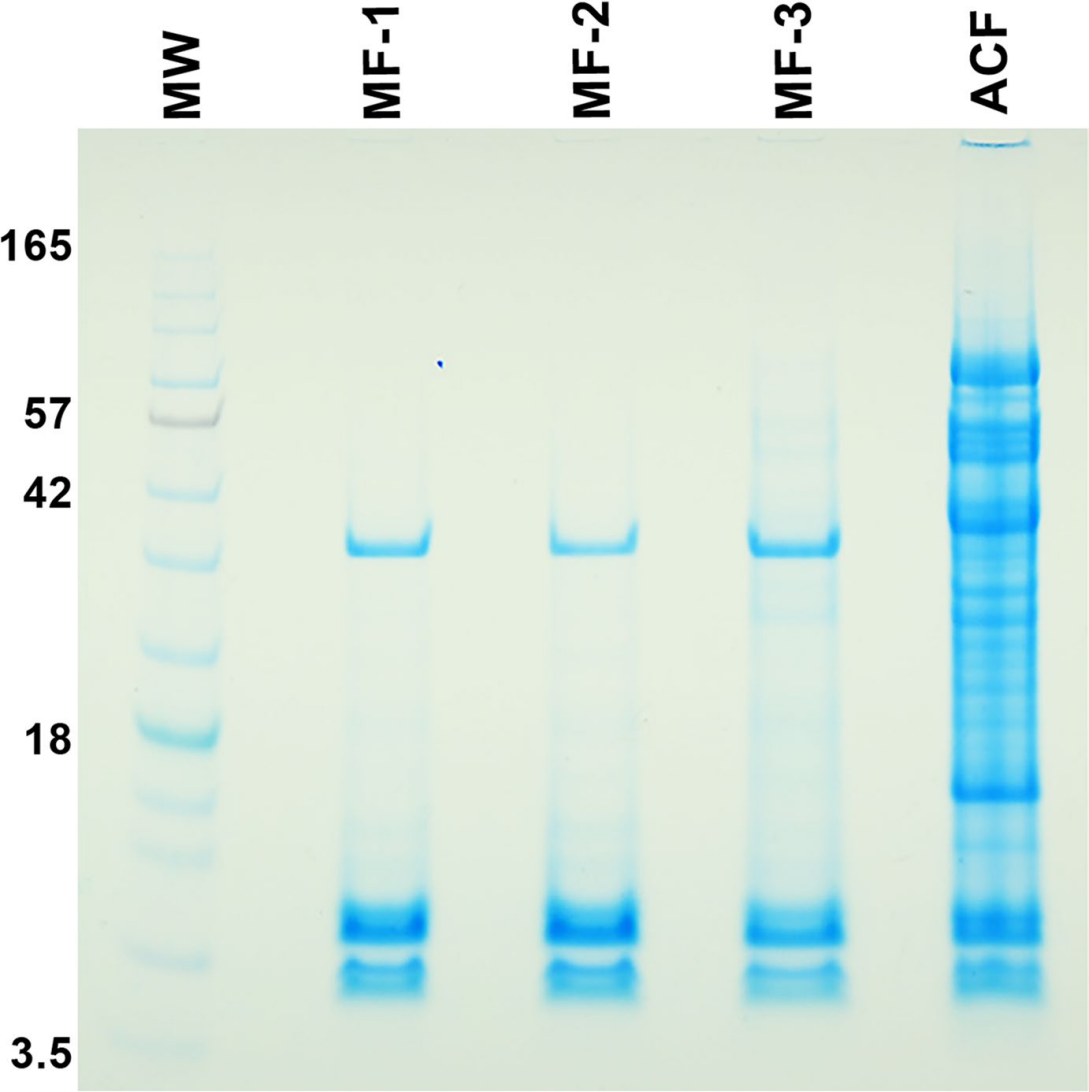
Sodium dodecyl sulfate–polyacrylamide gel with 20 µL of either megagametophyte fluid (MF) or archegonial chamber fluid (ACF) per lane. ACF, MF-1, MF-2, MF-3 were all collected from ovules of one individual of *Cycas revoluta*. A molecular ladder of standard proteins is to the left

According to Bradford analysis, MF had less protein than ACF: MF had an average of less than 0.5 µg µl^−1^ protein, whereas ACF had close to 2.5 µg µl^−1^ (Fig. 6). There was also a corresponding increase in the number of bands of proteins separated by SDS-PAGE (Fig. 7).

**Fig. 8:**
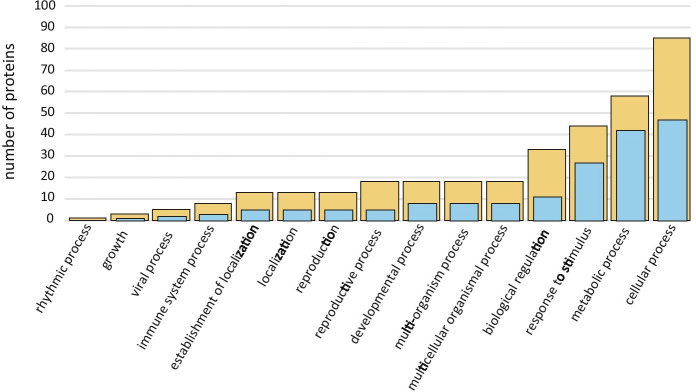
Histogram of number of proteins involved in Biological Functions categorized by Gene Ontology Annotations. Blue bars: proteins found in both megagametophyte fluid (MF) and archegonial chamber fluid (ACF, yellow). Yellow bars: proteins found only in ACF alone

We identified over 150 proteins by SDS-PAGE separation followed by processing for HPLC–MS/MS analysis (Table 1). The first fluid to be released, MF, had 55 proteins, all of which were also found in ACF, i.e. MF did not have any unique proteins. ACF was much richer, having another 90 unique proteins not found in MF. When these were associated with gene ontology annotations (GOA) the proteins in both samples—even though they greatly differed in number—were distributed similarly among the various GO processes. In short, ACF proteins were of similar functions to MF proteins, only more numerous. There was overall similarity in the relative numbers of proteins found in the Biological Function categories in both ACF and MF (Fig. 8), i.e. ACF had more of the same types of proteins that were found in MF. Cellular and metabolic processes were dominant, and to a slightly lesser degree stimulus response and biological regulation. Examples of proteins involved in cellular processes were proteins involved in glycolytic processes, e.g. fructose-bisphosphate aldolase 5, proteolysis, e.g. proteasome subunit beta type-2-A, and chaperone-mediated protein folding, e.g. heat shock protein 83. Examples of proteins involved in metabolic processes were proteins involved in gluconeogenesis, e.g. glucose-6-phosphate isomerase 1, hydrogen peroxide catabolism, e.g. l-ascorbate peroxidase, and translation, e.g. 60S ribosomal protein L11-1. Examples of proteins involved in stimulus responses were proteins involved in defense responses to bacteria, e.g. heat shock protein 90–1, responses to stresses such as heat, e.g. heat shock 70 kDa protein 18. Fewer proteins were involved in reproduction, development or growth. The overall conclusion in terms of biological function was that this extracellular fluid had a large complement of proteins that were released cytoplasmic contents.

**Table 1:** Alphabetically ordered list of proteins identified in archegonial chamber fluid (ACF) and megagametophyte fluid (MF, indicated by + sign) of *Cycas revoluta*, including Uniprot access numbers and associated organisms

N^o^	ACF proteins	Access no	MF	Organism
1	14–3-3-Like protein	14332_ARATH		*Arabidopsis thaliana*
2	14–3-3-Like protein	14312_ARATH	+	*Arabidopsis thaliana*
3	14–3-3-Like protein	14333_ARATH	+	*Arabidopsis thaliana*
4	14–3-3-Like protein	1433E_TOBAC	+	*Nicotiana tabacum*
5	18.0 kDa Class I heat shock protein	HSP12_DAUCA		*Daucus carota*
6	26S Protease regulatory subunit 6B homolog	PRS6B_ARATH		*Arabidopsis thaliana*
7	26S Proteasome non-ATPase regulatory subunit 1 homolog B	PSD1B_ARATH		*Arabidopsis thaliana*
8	26S Proteasome non-ATPase regulatory subunit 3 homolog A	PSD3A_ARATH		*Arabidopsis thaliana*
9	26S Proteasome non-ATPase regulatory subunit 6 homolog	PSMD6_ARATH		*Arabidopsis thaliana*
10	26S Proteasome non-ATPase regulatory subunit 7 homolog A	PSD7A_ARATH		*Arabidopsis thaliana*
11	40S Ribosomal protein S14-1	RS141_ARATH		*Arabidopsis thaliana*
12	40S Ribosomal protein S15a-1	R15A1_ARATH		*Arabidopsis thaliana*
13	40S Ribosomal protein S15a-3	R15A3_ARATH		*Arabidopsis thaliana*
14	40S Ribosomal protein S18	RS18_ARATH		*Arabidopsis thaliana*
15	40S Ribosomal protein S25-2	RS252_ARATH		*Arabidopsis thaliana*
16	40S Ribosomal protein S27-1	RS271_ARATH		*Arabidopsis thaliana*
17	40S Ribosomal protein S3-3	RS33_ARATH		*Arabidopsis thaliana*
18	40S Ribosomal protein S5-1	RS51_ARATH		*Arabidopsis thaliana*
19	40S Ribosomal protein S9-1	RS91_ARATH	+	*Arabidopsis thaliana*
20	5-Methyltetrahydropteroyltriglutamate–homocysteine methyltransferase	METE_PLESU	+	*Plectranthus scutellarioides*
21	5-Oxoprolinase	OPLA_ARATH		*Arabidopsis thaliana*
22	60S Ribosomal protein L10a-2	R10A2_ARATH		*Arabidopsis thaliana*
23	60S Ribosomal protein L11-1	RL111_ARATH	+	*Arabidopsis thaliana*
24	60S Ribosomal protein L14-1	RL141_ARATH	+	*Arabidopsis thaliana*
25	60S Ribosomal protein L26-2	RL262_ARATH		*Arabidopsis thaliana*
26	60S Ribosomal protein L3-2	RL32_ARATH		*Arabidopsis thaliana*
27	60S Ribosomal protein L4	RL4_PRUAR		*Prunus armeniaca*
28	60S Ribosomal protein L4-1	RL4A_ARATH		*Arabidopsis thaliana*
29	60S Ribosomal protein L7-3	RL73_ARATH		*Arabidopsis thaliana*
30	60S Ribosomal protein L9-2	RL92_ARATH		*Arabidopsis thaliana*
31	ABC transporter E family member	AB2E_ARATH		*Arabidopsis thaliana*
32	Actin	ACT_PINCO		*Pinus contorta*
33	Actin	ACT7_ARATH		*Arabidopsis thaliana*
34	Adenosylhomocysteinase 1	SAHH1_ARATH	+	*Arabidopsis thaliana*
35	ADP-ribosylation factor 1	ARF1_ARATH		*Arabidopsis thaliana*
36	Aldo–keto reductase family 4 member	AKRCA_ARATH	+	*Arabidopsis thaliana*
37	Aminopeptidase	APM1_ARATH		*Arabidopsis thaliana*
38	Aminopeptidase	APM1A_ORYSJ		*Oryza sativa* ssp *japonica*
39	Arginine–tRNA ligase, chloroplastic/mitochondrial	SYRM_ARATH		*Arabidopsis thaliana*
40	Aspartate aminotransferase	AAT2_ARATH	+	*Arabidopsis thaliana*
41	Aspartate aminotransferase	AATC_ORYSJ	+	*Oryza sativa* ssp *japonica*
42	Aspartate aminotransferase	AAT5_ARATH		*Arabidopsis thaliana*
43	Auxin transport protein BIG	BIG_ORYSJ [2]		*Oryza sativa* ssp *japonica*
44	Auxin-induced protein	A115_TOBAC		*Nicotiana tabacum*
45	Calmodulin	CALM_WHEAT		*Triticum aestivum*
46	Cell division control protein 48 homolog A	CD48A_ARATH		*Arabidopsis thaliana*
47	Cell division control protein 48 homolog D	CD48D_ARATH		*Arabidopsis thaliana*
48	Cell division cycle protein 48 homolog	CDC48_SOYBN	+	*Glycine max*
49	Chaperone protein ClpB1	CLPB1_ARATH		*Arabidopsis thaliana*
50	Chaperonin 60 subunit beta 1	CPNB1_ARATH		*Arabidopsis thaliana*
51	Clathrin heavy chain 1	CLAH1_ARATH		*Arabidopsis thaliana*
52	Clathrin heavy chain 1	CLH1_ORYSJ		*Oryza sativa* ssp *japonica*
53	Cluster of 40S ribosomal protein S5 (Fragment)	RS5_CICAR		*Cicer arietenum*
54	Cluster of 60S ribosomal protein L11-1	RL111_ARATH	+	*Arabidopsis thaliana*
55	Cluster of 60S ribosomal protein L7a-2	RL7A2_ORYSJ		*Oryza sativa* ssp *japonica*
56	Cluster of Malate dehydrogenase, cytoplasmic	MDHC_BETVU	+	*Beta vulgaris*
57	COP9 signalosome complex subunit 4	CSN4_ARATH		*Arabidopsis thaliana*
58	Cullin-1	CUL1_ARATH		*Arabidopsis thaliana*
59	Cullin-associated NEDD8-dissociated protein 1	CAND1_ARATH		*Arabidopsis thaliana*
60	Cytosolic isocitrate dehydrogenase [NADP]	ICDHC_ARATH	+	*Arabidopsis thaliana*
61	DNA damage-binding protein 1a	DDB1A_ARATH		*Arabidopsis thaliana*
62	DNA damage-binding protein 1b	DDB1B_ARATH		*Arabidopsis thaliana*
63	Elongation factor 1-alpha	EF1A_MANES	+	*Manihot esculentum*
64	Elongation factor 1-alpha 2	EF1A2_ARATH		*Arabidopsis thaliana*
65	Elongation factor 2	EF2_BETVU	+	*Beta vulgaris*
66	Elongation factor 2	EF2_ARATH		*Arabidopsis thaliana*
67	Eukaryotic initiation factor 4A-1	IF4A1_ORYSJ	+	*Oryza sativa* ssp *japonica*
68	Eukaryotic peptide chain release factor subunit 1–1	ERF1X_ARATH		*Arabidopsis thaliana*
69	Fructose-bisphosphate aldolase 5	ALFC5_ARATH	+	*Arabidopsis thaliana*
70	Fructose-bisphosphate aldolase 8	ALFC8_ARATH	+	*Arabidopsis thaliana*
71	Glucose-6-phosphate isomerase 1, chloroplastic	G6PIP_ARATH	+	*Arabidopsis thaliana*
72	Glucose-6-phosphate isomerase, cytosolic	G6PI_ARATH		*Arabidopsis thaliana*
73	Glucose-6-phosphate isomerase, cytosolic A	G6PIA_ORYSJ	+	*Oryza sativa* ssp *japonica*
74	Glutathione S-transferase U20	GSTUK_ARATH	+	*Arabidopsis thaliana*
75	Glyceraldehyde-3-phosphate dehydrogenase	G3PC_GINBI	+	*Ginkgo biloba*
76	Guanosine nucleotide diphosphate dissociation inhibitor 1	GDI1_ARATH	+	*Arabidopsis thaliana*
77	Heat shock 70 kDa protein	HSP70_HYDVU]		*Hydra vulgaris*
78	Heat shock 70 kDa protein 10, mitochondrial	HSP7J_ARATH		*Arabidopsis thaliana*
79	Heat shock 70 kDa protein 15	HSP7P_ARATH		*Arabidopsis thaliana*
80	Heat shock 70 kDa protein 18	HSP7N_ARATH	+	*Arabidopsis thaliana*
81	Heat shock 70 kDa protein 3	HSP7C_ARATH	+	*Arabidopsis thaliana*
82	Heat shock 70 kDa protein, mitochondrial	HSP7M_PHAVU		*Phaseolus vulgaris*
83	Heat shock protein 83	HSP83_IPONI	+	*Ipomoea nil*
84	Heat shock protein 90–1	HS901_ARATH	+	*Arabidopsis thaliana*
85	Heat shock protein 90–4	HS904_ARATH	+	*Arabidopsis thaliana*
86	Heat shock protein 90–6, mitochondrial	HS906_ARATH		*Arabidopsis thaliana*
87	Hsp70-Hsp90 organizing protein 3	HSOP3_ARATH	+	*Arabidopsis thaliana*
88	Importin subunit beta-1	IMB1_ARATH	+	*Arabidopsis thaliana*
89	Inositol hexakisphosphate and diphosphoinositol-pentakisphosphate kinase VIP1	VIP1L_ARATH		*Arabidopsis thaliana*
90	Isocitrate dehydrogenase [NADP]	IDHC_SOLTU	+	*Solanum tuberosum*
91	Isoleucine–tRNA ligase, cytoplasmic	SYIC_ARATH		*Arabidopsis thaliana*
92	l-arabinokinase	ARAK_ARATH		*Arabidopsis thaliana*
93	l-ascorbate peroxidase 1	APX1_ARATH	+	*Arabidopsis thaliana*
94	l-ascorbate peroxidase 2	APX2_ARATH	+	*Arabidopsis thaliana*
95	l-ascorbate peroxidase 3	APX3_ARATH		*Arabidopsis thaliana*
96	Leucine aminopeptidase 2	AMPL2_ORYSJ	+	*Oryza sativa* ssp *japonica*
97	Malate dehydrogenase, chloroplastic	MDHP_ARATH	+	*Arabidopsis thaliana*
98	NADP-dependent malic enzyme 1	MAOP1_ARATH	+	*Arabidopsis thaliana*
99	Nuclear pore complex protein NUP133	NU133_ARATH		*Arabidopsis thaliana*
100	Organellar oligopeptidase A, chloroplastic/mitochondrial	OOPDA_ARATH		*Arabidopsis thaliana*
101	Peroxisomal isocitrate dehydrogenase [NADP]	ICDHX_ARATH	+	*Arabidopsis thaliana*
102	Probable cytosolic oligopeptidase A	COPDA_ARATH		*Arabidopsis thaliana*
103	Probable histone chaperone ASF1A	ASF1A_ARATH		*Arabidopsis thaliana*
104	Probable mediator of RNA polymerase II transcription subunit 37b	MD37B_ARATH		*Arabidopsis thaliana*
105	Probable voltage-gated potassium channel subunit beta	KCAB_ARATH		*Arabidopsis thaliana*
106	Proliferating cellular nuclear antigen 1	PCNA1_ARATH	+	*Arabidopsis thaliana*
107	Proteasome subunit alpha type-3	PSA3_ARATH	+	*Arabidopsis thaliana*
108	Proteasome subunit alpha type-5-A	PSA5A_ARATH	+	*Arabidopsis thaliana*
109	Proteasome subunit alpha type-6	PSA6_SOYBN	+	*Glycine max*
110	Proteasome subunit alpha type-6-A	PSA6A_ARATH		*Arabidopsis thaliana*
111	Proteasome subunit beta type-2-A	PSB2A_ARATH	+	*Arabidopsis thaliana*
112	Proteasome subunit beta type-3-A	PSB3A_ARATH	+	*Arabidopsis thaliana*
113	Proteasome subunit beta type-6	PSB6_ARATH	+	*Arabidopsis thaliana*
114	Protein EXPORTIN 1A	XPO1A_ARATH		*Arabidopsis thaliana*
115	Protein ILITYHIA	ILA_ARATH		*Arabidopsis thaliana*
116	Puromycin-sensitive aminopeptidase	PSA_ARATH	+	*Arabidopsis thaliana*
117	Pyrophosphate–fructose 6-phosphate 1-phosphotransferase subunit beta	PFPB_SOLTU		*Solanum tuberosum*
118	Pyruvate decarboxylase 2	PDC2_ARATH		*Arabidopsis thaliana*
119	Ras-related protein Rab11C	RB11C_LOTJA		*Lotus japonicus*
120	Ras-related protein RABA1f	RAA1F_ARATH		*Arabidopsis thaliana*
121	Ras-related protein RABA2c	RAA2C_ARATH		*Arabidopsis thaliana*
122	Ras-related protein RABA3	RABA3_ARATH		*Arabidopsis thaliana*
123	Ras-related protein RABA4d	RAA4D_ARATH		*Arabidopsis thaliana*
124	Ras-related protein RABB1c	RAB1C_ARATH		*Arabidopsis thaliana*
125	Ras-related protein RGP1	RLGP1_ORYSJ		*Oryza sativa* ssp *japonica*
126	RuBisCO large subunit-binding protein subunit beta, chloroplastic	RUBB_PEA		*Pisum sativum*
127	RuvB-like protein 1	RIN1_ARATH		*Arabidopsis thaliana*
128	Serine hydroxymethyltransferase 4	GLYC4_ARATH		*Arabidopsis thaliana*
129	Serine/threonine-protein phosphatase	2AAB_ARATH		*Arabidopsis thaliana*
130	Serine/threonine-protein phosphatase PP1 isozyme 3	PP13_ARATH		*Arabidopsis thaliana*
131	SKP1-like protein 1A	SKP1A_ARATH	+	*Arabidopsis thaliana*
132	Small ubiquitin-related modifier 1	SUMO1_ARATH		*Arabidopsis thaliana*
133	T-complex protein 1 subunit alpha	TCPA_ARATH		*Arabidopsis thaliana*
134	T-complex protein 1 subunit beta	TCPB_ARATH		*Arabidopsis thaliana*
135	T-complex protein 1 subunit delta	TCPD_ARATH	+	*Arabidopsis thaliana*
136	T-complex protein 1 subunit epsilon	TCPE_ARATH		*Arabidopsis thaliana*
137	T-complex protein 1 subunit eta	TCPH_ARATH		*Arabidopsis thaliana*
138	T-complex protein 1 subunit gamma	TCPG_ARATH		*Arabidopsis thaliana*
139	T-complex protein 1 subunit theta	TCPQ_ARATH		*Arabidopsis thaliana*
140	T-complex protein 1 subunit zeta 1	TCPZA_ARATH		*Arabidopsis thaliana*
141	Tubulin alpha-1 chain	TBA1_HORVU		*Hordeum vulgare*
142	Tubulin alpha-6 chain	TBA6_ARATH		*Arabidopsis thaliana*
143	Ubiquitin carboxyl-terminal hydrolase 12	UBP12_ARATH	+	*Arabidopsis thaliana*
144	Ubiquitin carboxyl-terminal hydrolase 6	UBP6_ARATH		*Arabidopsis thaliana*
145	Ubiquitin-activating enzyme E1 2	UBE12_ARATH		*Arabidopsis thaliana*
146	Ubiquitin-conjugating enzyme E2 35	UBC35_ARATH	+	*Arabidopsis thaliana*
147	Ubiquitin-NEDD8-like protein RUB1	RUB1_ARATH		*Arabidopsis thaliana*
148	UDP-D-apiose/UDP-D-xylose synthase 1	AXS1_ARATH	+	*Arabidopsis thaliana*
149	UDP-glucose 4-epimerase 5	UGE5_ARATH		*Arabidopsis thaliana*
150	UDP-glucuronic acid decarboxylase 3	UXS3_ARATH		*Arabidopsis thaliana*
151	V-type proton ATPase subunit B 2 (Fragment)	VATB2_GOSHI	+	*Gossypium hirsutum*
152	V-type proton ATPase subunit B2	VATB2_ARATH	+	*Arabidopsis thaliana*
153	Valine–tRNA ligase, mitochondrial 1	SYVM1_ARATH		*Arabidopsis thaliana*
154	Villin-3	VILI3_ARATH		*Arabidopsis thaliana*

**Fig. 9:**
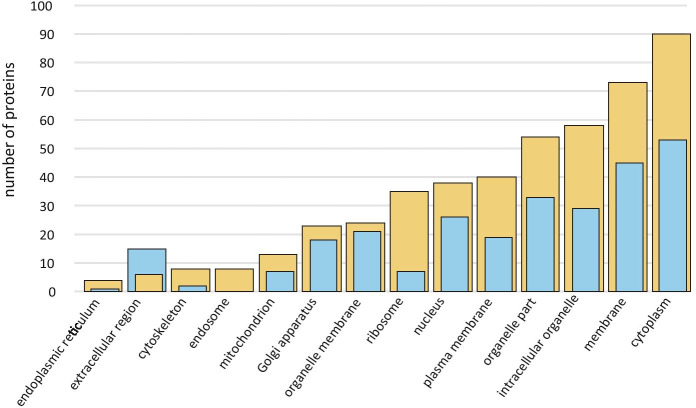
Histogram of number of proteins involved in Cellular Components categorized by Gene Ontology Annotations. Blue bars: proteins found in both megagametophyte fluid (MF) and archegonial chamber fluid (ACF). Yellow bars: proteins found only in ACF alone

This impression was further reinforced by the GOA analysis of the cellular components. Best represented were cytoplasmic proteins, such as malate dehydrogenases and proteasome subunit proteins, as well as intracellular organelle-related proteins, such as those associated with chloroplasts, e.g. chloroplast malate dehydrogenase, or with Golgi, e.g. 14-3-3 proteins, or with membranes, e.g. importin subunit beta-1. Less well represented were mitochondrial proteins, e.g. glyceraldehyde-3-phosphate dehydrogenase, and ribosomal proteins, such as 60 s ribosomal protein L11-1, implying that these proteins were from cells that were not particularly metabolically or transcriptionally active. The cellular component protein fraction was similarly distributed: ACF had more of the same types of protein whether they were cytoplasmic, organellar or membrane proteins (Fig. 9). One notable exception was the presence of more extracelluar region proteins in the MF than the ACF, which was the reverse of the general trend of there being more proteins in the ACF than the MF.

**Fig. 10:**
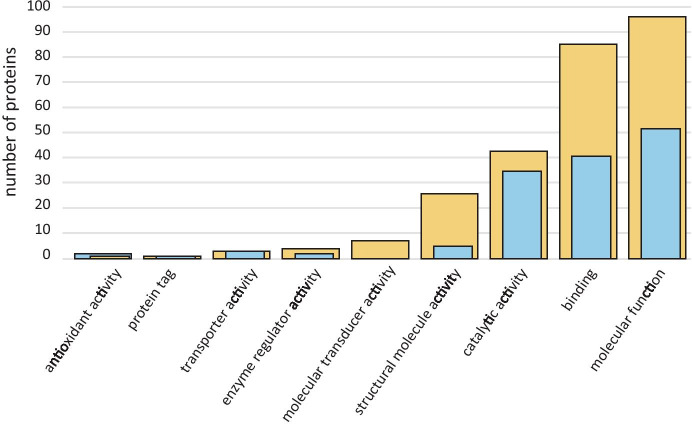
Histogram of number of proteins involved in Molecular Functions categorized by Gene Ontology Annotations. Blue bars: proteins found in both megagametophyte fluid (MF) and archegonial chamber fluid (ACF). Yellow bars: proteins found only in ACF alone

In terms of molecular functions, the GOA protein profile included general functions such as ATP binding, e.g. V-type proton ATPase subunit B2, and NAD binding, e.g. NADP-dependent malic enzyme. Other well represented molecular functions were binding, such as DNA binding, e.g. proliferating cell nuclear antigen, and catalytic activity, such as threonine-type endopeptidase activity, e.g. proteasome subunit alpha type 6 (Fig. 10).

### Protein Expression

**Fig. 11:**
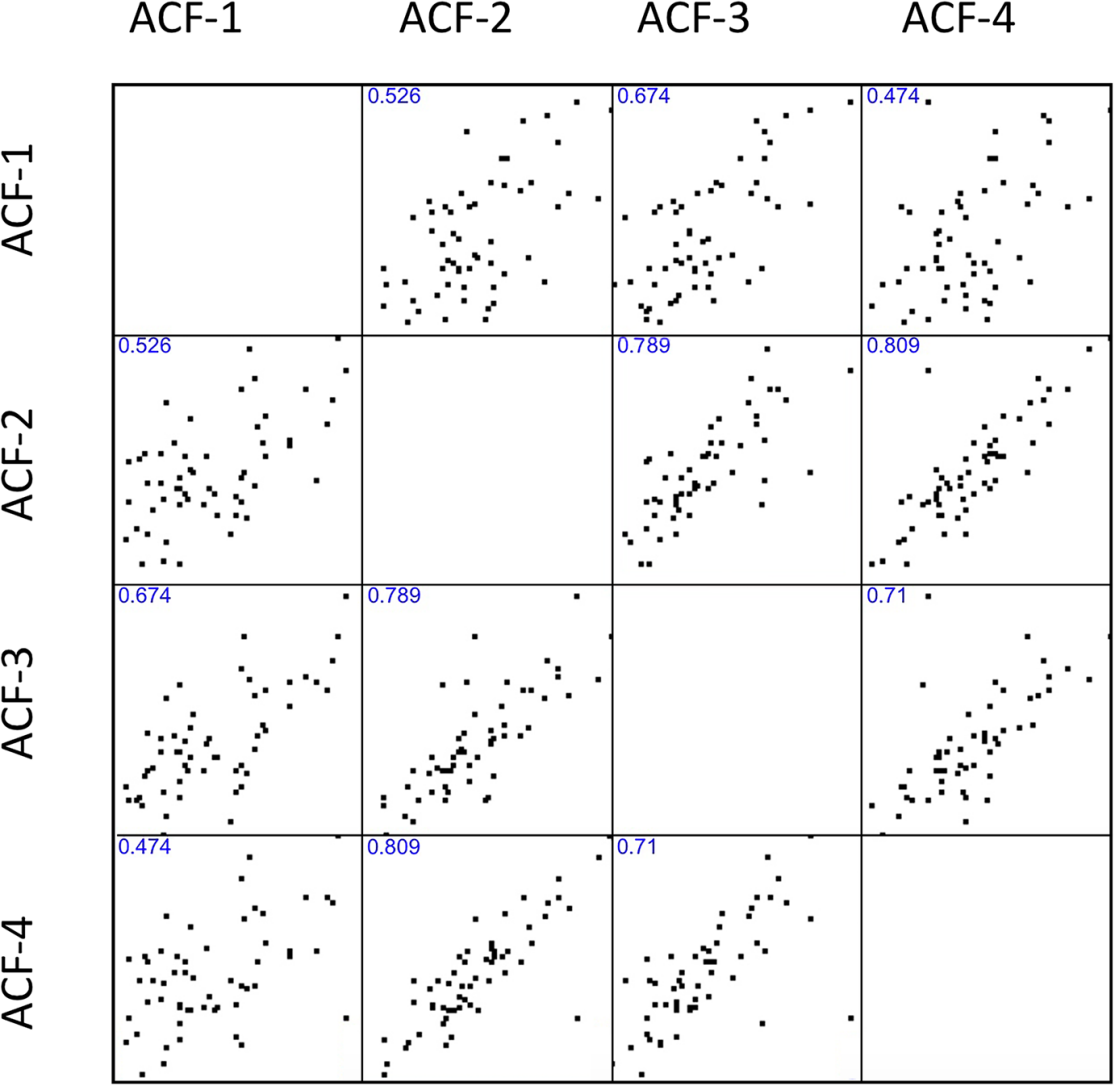
Spearman coefficient analysis of protein expression in four individuals’ archegonial chamber fluids (ACF)

Label-free quantitation using liquid–liquid extraction and processing followed with standardized amounts of protein followed by HPLC–MS/MS analysis of MF and ACF fluids, was used to identify a subset of 53 proteins found across all samples (Supporting Information Table S2). Archegonial chamber fluids, for example, did not differ in its protein expression (Fig. 11). Spearman’s coefficient analysis showed that there was, generally, a positive relationship between individuals as well as between types of fluids, i.e. in this filtered selection of proteins, the most commonly expressed proteins were universally commonly expressed. Pearson’s profile analysis as shown in the heat map showed that in relative terms, only a small number of proteins showed greater than twofold expression differences (not shown). Among ACF proteins these included glucose-6-phosphate isomerase and peptidylprolyl isomerase, two important metabolic enzymes. In MFs, three enzymes were found with an expression that was more than twofold greater than their expression in ACF. These included two metabolic enzymes–aldolase and glyceraldehyde-3-phosphate dehydrogenase–and a defense enzyme, chitinase A.

### Pollination Drop Protein Profile

A total of 35 proteins were identified by liquid–liquid extraction and processing followed by HPLC–MS/MS analysis. There were 23 proteins that met our criteria, but could not be identified because of current limitations in databases. Of the remaining dozen, they divided into three categories: five were related to defense and stress responses, five were cell wall or carbohydrate modifying enzymes and three had other diverse functions (Table 2). None of these were found in the sexual fluids secreted by the megagametophyte.

**Table 2:** Alphabetical list of proteins identified in the pollination drop of *Cycas revoluta,* including Uniprot access numbers and associated organisms

N^o^	Pollination drop protein	Access no	Organism
1	Amine oxidase	A0A059B3H6_EUCGR	*Eucalyptus grandis*
2	Amine oxidase	A0A0D6QWY4_ARACU	*Araucaria cunninghamii*
3	Cysteine proteinase inhibitor	CYTI_VIGUN	*Vigna unguiculata*
4	Dirigent-like protein	DIRLP_PINHA	*Pinus halepensis*
5	KDEL-tailed cysteine endopeptidase	CEP2_ARATH	*Arabidopsis thaliana*
6	PR-4 protein	Q9M7D9_PEA	*Pisum sativum*
7	Thioredoxin	TRH41_ORYSJ	*Oryza sativa* ssp *japonica*
8	Alpha-galactosidase	AGAL_COFAR	*Coffea arabica*
9	Beta-galactosidase	BGAL5_ORYS	*Oryza sativa* ssp *japonica*
10	Glycerophosphodiester Phosphodiesterase	GPDL3_ARATH	*Arabidopsis thaliana*
11	LRR-extensin-like protein	G7L8Y9_MEDTR	*Medicago truncatula*
12	Beta-xylosidase	K4HQ36_9ROSA	*Prunus salicina*
13	Caleosin	B8XX15_CYCRE	*Cycas revoluta*
14	Auxin-responsive protein IAA12	IAA12_ORYSJ	*Oryza sativa* ssp *japonica*
15	Cannibidiiolic acid synthase	CBDAS_CANSA	*Cannabis sativa*
16	Probable terpene synthase 13	TPS13_RICCO	*Ricinus communis*

### Carbohydrate Analysis

**Fig. 12:**
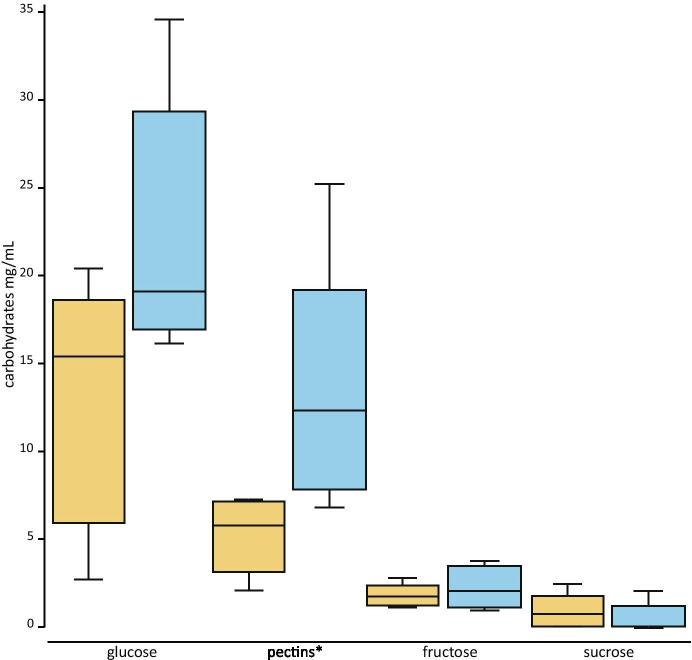
Boxplot of carbohydrate concentrations (mg/mL) of megagametophyte fluid (MF, blue) and archegonial chamber fluid (ACF, yellow) of *Cycas revoluta*. Pectin concentrations are in galacturonic equivalents (mg/mL)

The most generally abundant sugar was glucose. Compared to MF, archegonial fluid had a lower concentration of glucose (Fig. 12, Supporting Information Table S3). Sucrose and fructose were low in abundance in both fluids. In addition to these sugars, pectin was also present. Similar to glucose, it was found at higher concentrations in the MF than in the ACF (Fig. 12).

### Amino Acid Analysis

**Fig. 13:**
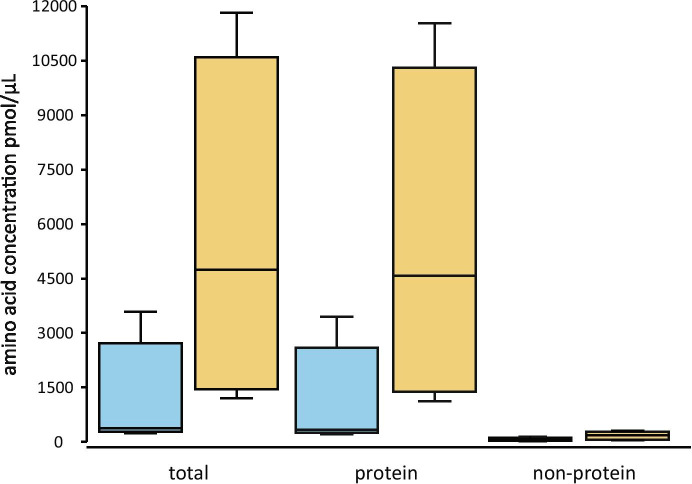
Boxplot of amino acid concentrations (pmol/µL) of megagametophyte fluid (MF, blue) and archegonial chamber fluid (ACF, yellow) of *Cycas revoluta*

The highest concentrations of amino acids were found in ACF (Fig. 13). Of these, the highest concentrations were generally protein amino acids, of which glutamic acid, alanine and proline (Supporting Information Table S4) had the highest concentrations. The non-protein acids were much less abundant (Fig. 13). Of these, beta-alanine and ornithine had the highest concentrations (Supporting Information Table﻿ S4).

### pH

Megagametophyte fluid had a slightly acidic pH (5.9 ± 1.2), whereas that of ACF was more alkaline (8.4 ± 1.3). The emission of substances into MF from the archegonium appears to have raised the pH significantly, *F*(1, 56) = 12.97, *p* < 0.001. There was also a significant plant effect on pH of MF and ACF, *F*(6, 49) = 6.88, *p* < 0.001. Plant based variation contributes to the wide ranges: ACF, pH 6.5–9.1; MF, pH 5.2–8.4.

### Osmotic Concentration

Megagametophyte fluid had a much lower median osmotic concentration (678 mOsm) than that of ACF (918 mOsm; Fig.﻿ 14). The addition of numerous substances released from the one or two archegonia that opened following MF exposure resulted in an increase in the final osmotic concentration of ACF.

**Fig. 14:**
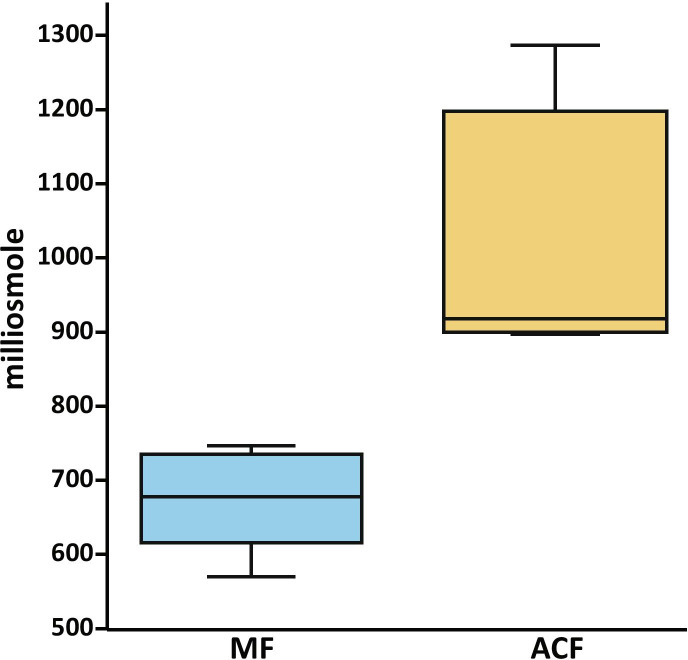
Boxplot of osmotic potential in milliosmole﻿ of megagametophyte fluid (MF, blue) and archegonial chamber fluid (ACF, yellow) of *Cycas revoluta*

## Discussion

Analysis of these fluids’ composition suggest hitherto undescribed mechanisms in seed plant reproduction. Sexual fluids released by the ovule during pollination and fertilization differ in their origins, functions and composition. In zoidogamous gymnosperms, the source of these fluids are the internal tissues of the ovule itself. Pollination drops, megagametophyte (MF) and archegonial chamber fluids (ACF) are complex; they have stage-specific protein profiles. Fluids such as MF and ACF are also rich in amino acids, but low in carbohydrates compared to pollination drops or floral nectar. This leads us to conclude that these fluids have reproductive functions beyond simple pollen capture and transport, in the case of pollination drops, or sperm transport, in the case of ACF and MF. Sexual fluids represent a form of female control over male development, including induction of pollen germination by pollination drops and release of sperm from pollen tubes by internal ovule fluids.

Megagametophyte fluid and archegonial chamber fluid did not have any protein functions in common with pollination drops. Protein profiles are of two distinct kinds. The pollination drop proteome represents a secretome, that is secreted enzymes whose functions are defense and carbohydrate-modification (Pirone-Davis et al., 2016; von Aderkas et al., 2018). The reported results list a few dozen proteins. In comparison, the more than 150 identified proteins found in MF and ACF profiles represent a degradome, that is proteins released from dying cells. As pollination precedes fertilization by nearly 3 months, we will discuss the related proteins in that order.

Pollination-related adaptations involve proteins. Because gymnosperm ovules are exposed to the outside, ovule surfaces have morphological and physiological adaptations that facilitate pollen capture and pollen germination (Leslie & Boyce, 2012; Little et al., 2014). Our results confirm that proteins in the pollination drop have limited functions (Gelbart & von Aderkas, 2002), i.e. defense against microbial pathogens and modification of carbohydrates and/or cell walls. These functions are conservative across cycads (Prior et al., 2018), conifers (Coulter et al., 2012), *Ginkgo* (Little et al., 2014) and gnetophytes (Hou et al., 2019).

By comparison, MF and ACF proteins are unable to function outside of the cell. The proteome of these two sexual fluids represents a broad range of functions, including gluconeogenesis, translation, and peroxide catabolism, but as they are washed from dying cells into the apoplast, the cellular infrastructure that facilitates reactions by concentrating reactants, enzymes and products is lost. These proteins, as GOA annotations show, originated from a broad suite of organelles, including mainly plastids, Golgi apparatus and ribosomes. We detected very few proteins associated with endoplasmic reticulum, an organelle that has been reported as having significant activity during megagametophyte storage reserve accumulation in the cycad *Encephelartos natalensis* (Woodenberg et al., 2010). We also did not find proteins with GO annotations linked to nutrient reservoir activity, or transport activity. Comparatively speaking, there was a diversity of programmed cell death (PCD)-linked proteins, such as proteasome and ubiquitination-linked proteins. PCD plays a significant role in archegonial chamber formation: in *Gingko biloba*, the nucellar portion of the archegonial chamber is formed by regulated cell breakdown (Li et al., 2007). The relatively high concentrations of cellular proteins found in MF and ACF are due to apoptosis-related events associated with breakdown of epidermal cells and not to necrotic, damage-related release as might occur due to infestation or wounding. Further support comes from the label-free quantitation experiment in which several proteins are found with similar expression levels in all samples of MF and ACF from all individuals sampled. This could be interpreted to mean that fluid release is the result of an evenly regulated mechanism.

A portion of the ACF is ejected material from the archegonia in response to MF fluid, triggering the two neck cells to divide, resulting in the formation of a canal into the archegonium. This canal not only provides access for sperm into the archegonium, but also allows material to flow out of the archegonium. This ejected material mixes with the fluid in the archegonial chamber. In the context of archegonial development, cellular content release is a fertilization-related adaptation that cycads share with free-sporing plants. During archegonium formation in these plants, egg and ventral canal cell are the last cells formed (Niklas & Kutschera, 2010). In the cycad *Encephelartos villosus,* ventral canal cell breakdown is followed by a surge of cytoplasm towards the neck (Steyn et al., 1996), which once ejected, allows sperm to enter. The wholesale dumping of cytoplasm into the apoplast, as also occurs during holocrine secretion during nectar formation (Vesprini et al., 2008), may be more than bulk deposit. For example, in other plants, ligand functions relating to the immune system have been found among compounds released by dying cells (Jones & Dangl, 2006). Chemotactic substances are likely to be found in the ACF, which may open the possibility of studying the molecular physiology of sperm in relation to isolated candidate molecules. In our study we did not find any proteins with GO annotations related to chemotaxis, but this does not rule out other compounds that may have ligand functions related to sperm attraction.

This study is the first to report release of carbohydrates from the surface of megagametophytes; carbohydrate release from archegonia has been previously described for ferns and mosses by Cao et al. (2017). Concentrations of sucrose and fructose were low in *C. revoluta*, but glucose concentrations were relatively high. The absence of sucrose in the swimming medium of cycad sperm suggests that changes to sperm experiments are needed. Pollen tubes will not burst on their own and water by itself is not sufficient to burst cycad pollen tubes (Takaso et al., 2013). Instead, solutions with sucrose concentrations greater than 10% (w/v) are needed to get pollen tubes to release their sperm (Hori & Miyamura, 1997), and to get archegonia to open their necks (Norstog, 1972). Unfortunately, sucrose solutions have been less than ideal for sperm, whose movements stop within five to ten minutes after exposure (Takaso et al., 2013). We would suggest that glucose replace sucrose. With the exception of pollination drops of Gnetales that have a relatively high sucrose concentration, sexual fluids of all other gymnosperms have low concentrations of sucrose (Nepi et al., 2017). Another abundant carbohydrate that we analyzed was pectin. Pectins are typically released either when cell walls are broken down (Anderson, 2016) or when the cell wall is ruptured during holocrine secretion (Nepi et al., 2009). Pectins are known from other reproductive systems. Studies of pectins in angiosperm reproduction have shown that de-esterified pectins located in the filiform apparatus are part of a complex regulatory mechanism involving nitric oxide (Duan et al., 2020).

A diversity of amino acids was also released before fertilization. This has not been previously reported for archegoniate plants. Total amino acid concentrations measured in MF and ACF were similar to other ovular secretions, such as pollination drops of *Ginkgo biloba* (Nepi et al., 2017). *Cycas revoluta* had high concentrations of glutamic acid, proline and alanine, which are commonly found in gymnosperm megagametophytes accumulating storage reserves (King & Gifford, 1997; Astarita et al., 2003). Among the non-protein amino acids, we noted the presence of ornithine. How compounds like ornithine that has a central position in plant nitrogen metabolism (Majumdar et al., 2015) affects cycad development is not known, but in other systems, ornithine released from dying cells is known to have biogenic effects on surrounding living cells (Medina et al*.*, 2020).

When proteins, carbohydrates and amino acids are released into the apoplast they directly affect properties of the solution. In the case of osmotic concentration, we can calculate the contribution of glucose, the dominant sugar: it is minor. Pectin’s contribution is similar, but in addition, pectin also has hygroscopic properties that may assist in extracting water from living cells. It may also influence the viscosity of the medium (Guimaraes et al., 2009). Proteins likely constitute the bulk of the osmotic force across the membranes of intact cells. They also probably drive the diffusion of water. This is because proteins that end up in the apoplast cannot pass back through intact membranes of living cells of the remaining megagametophyte cells, unlike glucose and amino acids. Protein concentrations may not be easily converted to osmotic concentrations, because proteins are able to dissociate into numerous ionic particles. Taken together, MF and ACF represent complex solutions. As a result, the solution in which cycad sperm swim is very different than, for example, rainwater in which fern or moss sperm swim. The generation of a solution rich in solutes for fertilization is a reproductive mechanism unique to zoidogamous seed plants.

If we compare our current analysis of carbohydrate and amino acid MF and ACF with previously published analyses of pollination drops carried out with the same methods and equipment, we can conclude that sexual fluids released by cycad ovules during fertilization are quite unlike those produced by gymnosperm ovules, including cycads, during pollination, and by angiosperms during floral nectar production (see non-metric multidimensional scaling (NMDS) plot—Supporting Information Fig. S1, Tables S3, S4).

Another mechanism unique to archegoniate plants involves the contents ejected from the archegonium. In cycads and in *Ginkgo biloba* this material lies in the immediate vicinity of the archegonium at the bottom of archegonial chambers. This rope-like flocculent mass has been referred to as an “egg projection” (Lee, 1955), a “protrusion” (Wang et al., 2014) and a “fertilization droplet” (Zhang et al., 2013). Similar to what we found in *C. revoluta*, Lee (1955) noted in *Ginkgo biloba* that the material is ejected into liquid already present in the archegonial chamber. Lee (1955) suggested that as the beating flagellae of sperm became entangled in this proteinaceous flocculent mass the sperm is drawn towards the neck of the archegonium. Unlike in other archegoniate plants, such as ferns and mosses, in which the ejected material is thought to have compounds that act as chemoattracts to sperm, the suggestion here is that physical entrapment is at work. However, one of us (TT) has observed sperm penetration of a cycad archegonium before it had ejected any materials. Zoidogamous sperm behaviour either in situ or ex situ, is extremely difficult to study and what we know is largely based on anecdotal asides in publications. The problem of how such a mechanism works is not likely to be resolved easily.

Male–female interactions are coordinated by the sexual fluids of *C. revoluta*. These interactions can be considered in terms of sporophyte and gametophtye. During pollination, the nucellus, a sporophytic portion of the ovule, secretes the pollination drop (O’Leary et al., 2004; Poulis et al. 2005). This maternal investment by the sporophyte regulates pollen germination (von Aderkas et al., 2018). During fertilization sexual fluids coordinate release of sperm from the microgametophyte, and provide a medium in which male gametes swim. These megagametophyte-derived fluids are produced in cycads (Chamberlain 1910; Lawson, 1926; Takaso et al., 2013) and *Ginkgo biloba* (Hirase, 1898; Lee, 1955; Friedman, 1987). Archegonia-derived fluids are known from both clades (Herzfeld, 1927; Takaso et al., 2013; Zhang et al., 2013). In conclusion, during zoidogamous reproduction there are stages in which the female produces complex sexual fluids that regulate the male. The complexity of sexual solutions, as revealed by our analyses, reveal new roles of the ovule-produced fluids in the evolution of sexual reproduction of seed plants.

## Supplementary Information

Below is the link to the electronic supplementary material.

Supplementary file1 (PDF 132 kb) Table S1 Locations of female plants of Cycas revoluta used in experiments. Table S2 ACF Proteins used in Figure 11 including their TREMBl (tr) or SwissProt (sp) FASTA identifiers. Table S3. Sugar concentrations (conc. mg/µl) of megagametophyte fluids and archegonial chamber fluids from four individuals of Cycas revoluta. Table S4. Amino acid concentrations (pmol/µl) of megagametophyte fluids and archegonial chamber fluids from four individuals of Cycas revoluta.

Supplementary file2 (DOCX 25 kb) Methods S1 Analytical methods used for proteomics, as well as carbohydrate and amino acid analysis.

Supplementary file3 (PDF 58 kb) Fig. S1 Non-metric multidimensional scaling plot of amino acid and carbohydrate composition.
