# Supplementary material for: Composition of Sexual Fluids in Cycas revoluta Ovules During Pollination and Fertilization
Source: Bot Rev. 2022 Jan 1;88(4):453–84. doi: 10.1007/s12229-021-09271-1 (PMC9726676; doi:10.1007/s12229-021-09271-1)
Supplement: Supplementary file 1 — Supplementary file1 (PDF 132 kb) Table S1 Locations of female plants of Cycas revoluta used in experiments. Table S2 ACF Proteins used in Figure 11 including their TREMBl (tr) or SwissProt (sp) FASTA identifiers. Table S3. Sugar concentrations (conc. mg/µl) of megagametophyte fluids and archegonial chamber fluids from four individuals of Cycas revoluta. Table S4. Amino acid concentrations (pmol/µl) of megagametophyte fluids and archegonial chamber fluids from four individuals of Cycas revoluta. [file 12229_2021_9271_MOESM1_ESM.pdf]

Table 1 Locations of female plants of *Cycas revoluta* used in experiments

Individual (female)	Latitude	Longitude
1	24.378577	123.752069
2	24.378635	123.752026
3	24.378646	123.752034
4	24.386117	123.747501
5	24.386117	123.747466
6	24.392939	123.755088
7	24.412411	123.805779
8	24.412433	123.805689
9	24.418353	123.799447
10	24.422714	123.775969
11	24.422769	123.776258
12	24.425347	123.765909
13	24.425372	123.790104
14	24.425459	123.794207
15	24.425459	123.790141
16	24.425466	123.790330
17	24.425504	123.765538
18	24.425521	123.765518
19	24.425637	123.765831
20	24.425641	123.765860
21	24.425662	123.793912
22	24.426464	123.793559
23	24.426692	123.729481
24	24.426882	123.764721

Table 2 ACF Proteins used in Figure 11 including their TREMBl (tr) or SwissProt (sp) FASTA identifiers

tr|A0A0D6R0F3|A0A0D6R0F3_ARACU alpha-1,2-Mannosidase OS=Araucaria cunninghamii OX=56994 PE=3 SV=1;tr|A0A0C9RRM4|A0A0C9RRM4_9SPER alpha-1,2-Mannosidase OS=Wollemia nobilis OX=56998 PE=3 SV=1

tr|A0A0D6QXU4|A0A0D6QXU4_ARACU Aspartate aminotransferase OS=Araucaria cunninghamii OX=56994 PE=4 SV=1

tr|B8LKC5|B8LKC5_PICSI Aspartate aminotransferase OS=Picea sitchensis OX=3332 PE=2 SV=1;tr|C0PSV4|C0PSV4_PICSI Aspartate aminotransferase OS=Picea sitchensis OX=3332 PE=2 SV=1

tr|A0A088DLF4|A0A088DLF4_CEPOL ATP synthase CF1 beta chain (Fragment) OS=Cephalotaxus oliveri OX=147282 GN=atpB PE=4 SV=1;tr|A0A088DJY5|A0A088DJY5_CEPFR ATP synthase CF1 beta chain (Fragment) OS=Cephalotaxus fortunei OX=66169 GN=atpB PE=4 SV=1;tr|Q8MFX2|Q8

tr|Q4W6L6|Q4W6L6_CYCRE Chitinase A OS=Cycas revoluta OX=3396 GN=crchiA PE=1 SV=1

tr|V5W8K7|V5W8K7_9SPER DHAR class glutathione S-transferase OS=Larix kaempferi OX=54800 GN=DHAR1 PE=2 SV=1;tr|L7S279|L7S279_PINTB DHAR class glutathione S-transferase OS=Pinus tabuliformis OX=88731 GN=DHAR1 PE=2 SV=1;tr|B2ZHM6|B2ZHM6_PINBU Dehydroascorbate

tr|A6YR00|A6YR00_PICSI Dirigent protein OS=Picea sitchensis OX=3332 PE=2 SV=1;tr|A6YQZ9|A6YQZ9_PICSI Dirigent protein OS=Picea sitchensis OX=3332 PE=2 SV=1

tr|A0A0D6R4B6|A0A0D6R4B6_ARACU Elongation factor Tu OS=Araucaria cunninghamii OX=56994 PE=3 SV=1

tr|Q8LK58|Q8LK58_METGY Fructose-bisphosphate aldolase OS=Metasequoia glyptostroboides OX=3371 PE=2 SV=1

tr|A9NMQ0|A9NMQ0_PICSI Fructose-bisphosphate aldolase OS=Picea sitchensis OX=3332 PE=2 SV=1;tr|A0A0C9RHN9|A0A0C9RHN9_9SPER Fructose-bisphosphate aldolase OS=Wollemia nobilis OX=56998 PE=3 SV=1;tr|A0A0D6RB10|A0A0D6RB10_ARACU Fructose-bisphosphate aldolase O

tr|A0A0D6QZF8|A0A0D6QZF8_ARACU Glucose-6-phosphate isomerase OS=Araucaria cunninghamii OX=56994 PE=3 SV=1;tr|A0A0C9S8P2|A0A0C9S8P2_9SPER Glucose-6-phosphate isomerase OS=Wollemia nobilis OX=56998 PE=3 SV=1

tr|E3V1T1|E3V1T1_PINSY Glutamate dehydrogenase (Fragment) OS=Pinus sylvestris OX=3349 GN=gdh PE=3 SV=1;tr|E3V1S5|E3V1S5_PINSY Glutamate dehydrogenase (Fragment) OS=Pinus sylvestris OX=3349 GN=gdh PE=3 SV=1;tr|D9IXC9|D9IXC9_PINPS Glutamate dehydrogenase OS=

tr|A0A1V1FYP3|A0A1V1FYP3_CRYJA Glyceraldehyde-3-phosphate dehydrogenase OS=Cryptomeria japonica OX=3369 GN=GAPDH PE=2 SV=1;tr|Q8LSX0|Q8LSX0_CRYJA Glyceraldehyde-3-phosphate dehydrogenase (Fragment) OS=Cryptomeria japonica OX=3369 GN=gapC PE=4 SV=1;tr|Q8LSW

sp|Q39769|G3PC_GINBI Glyceraldehyde-3-phosphate dehydrogenase, cytosolic OS=Ginkgo biloba OX=3311 PE=2 SV=1;tr|A0A0C9S6V5|A0A0C9S6V5_9SPER Glyceraldehyde-3-phosphate dehydrogenase OS=Wollemia nobilis OX=56998 PE=3 SV=1

tr|Q40924|Q40924_PSEMZ Luminal binding protein OS=Pseudotsuga menziesii OX=3357 GN=BiP PE=2 SV=2;tr|B8LS17|B8LS17_PICSI Uncharacterized protein OS=Picea sitchensis OX=3332 PE=2 SV=1;tr|Q9AVT8|Q9AVT8_PICAB Glucose regulated protein homolog 4 (Fragment) OS=P

tr|C0PQ46|C0PQ46_PICSI Malate dehydrogenase OS=Picea sitchensis OX=3332 PE=2 SV=1;tr|A9NVT9|A9NVT9_PICSI Malate dehydrogenase OS=Picea sitchensis OX=3332 PE=2 SV=1;tr|A9NP19|A9NP19_PICSI Malate dehydrogenase OS=Picea sitchensis OX=3332 PE=2 SV=1;tr|E3V293|

tr|A0A097PQY9|A0A097PQY9_GINBI Pectin lyase-like superfamily protein (Fragment) OS=Ginkgo biloba OX=3311 PE=2 SV=1;tr|K7P374|K7P374_PINCE Uncharacterized protein (Fragment) OS=Pinus cembra OX=58041 GN=2_7803_01 PE=3 SV=1;tr|K7P165|K7P165_PINMU Uncharacteri

tr|A9NUZ0|A9NUZ0_PICSI Peptidylprolyl isomerase OS=Picea sitchensis OX=3332 PE=2 SV=1

sp|P85347|PER2_CYCRE Peroxidase 2 (Fragment) OS=Cycas revoluta OX=3396 PE=1 SV=1;sp|P85433|PER7_CYCRE Peroxidase 7 (Fragment) OS=Cycas revoluta OX=3396 PE=1 SV=1;tr|A9NS12|A9NS12_PICSI Peroxidase OS=Picea sitchensis OX=3332 PE=2 SV=1

tr|A0A0C9S900|A0A0C9S900_9SPER Phosphoglycerate kinase OS=Wollemia nobilis OX=56998 PE=3 SV=1;tr|A0A0C9RW13|A0A0C9RW13_9SPER Phosphoglycerate kinase OS=Wollemia nobilis OX=56998 PE=3 SV=1;tr|A0A0D6R1F5|A0A0D6R1F5_ARACU Phosphoglycerate kinase OS=Araucaria

tr|A9P175|A9P175_PICSI Proliferating cell nuclear antigen OS=Picea sitchensis OX=3332 PE=2 SV=1

tr|A9NQT9|A9NQT9_PICSI Small ubiquitin-related modifier OS=Picea sitchensis OX=3332 PE=2 SV=1

tr|A0A0D6R8L6|A0A0D6R8L6_ARACU Superoxide dismutase [Cu-Zn] OS=Araucaria cunninghamii OX=56994 PE=3 SV=1;tr|A0A0D6R9V5|A0A0D6R9V5_ARACU Superoxide dismutase [Cu-Zn] OS=Araucaria cunninghamii OX=56994 PE=3 SV=1;tr|A0A0D6R0T3|A0A0D6R0T3_ARACU Superoxide dism

tr|A9NMW7|A9NMW7_PICSI Thioredoxin OS=Picea sitchensis OX=3332 PE=2 SV=1

tr|A0A0C9S6V2|A0A0C9S6V2_9SPER TSA: Wollemia nobilis Ref_Wollemi_Transcript_10465_2177 transcribed RNA sequence OS=Wollemia nobilis OX=56998 PE=3 SV=1;tr|A0A0D6R793|A0A0D6R793_ARACU Uncharacterized protein OS=Araucaria cunninghamii OX=56994 PE=3 SV=1

tr|A0A0C9S800|A0A0C9S800_9SPER TSA: Wollemia nobilis Ref_Wollemi_Transcript_12531_1084 transcribed RNA sequence OS=Wollemia nobilis OX=56998 PE=4 SV=1

tr|A0A0C9RT87|A0A0C9RT87_9SPER TSA: Wollemia nobilis Ref_Wollemi_Transcript_14058_2427 transcribed RNA sequence OS=Wollemia nobilis OX=56998 PE=3 SV=1

tr|A0A0C9RSZ1|A0A0C9RSZ1_9SPER TSA: Wollemia nobilis Ref_Wollemi_Transcript_14316_2269 transcribed RNA sequence OS=Wollemia nobilis OX=56998 PE=4 SV=1

tr|A0A0C9QP24|A0A0C9QP24_9SPER TSA: Wollemia nobilis Ref_Wollemi_Transcript_15289_2699 transcribed RNA sequence OS=Wollemia nobilis OX=56998 PE=3 SV=1

tr|A0A0C9RG66|A0A0C9RG66_9SPER TSA: Wollemia nobilis Ref_Wollemi_Transcript_28670_1179 transcribed RNA sequence OS=Wollemia nobilis OX=56998 PE=3 SV=1

tr|A0A0C9S9G7|A0A0C9S9G7_9SPER TSA: Wollemia nobilis Ref_Wollemi_Transcript_7751_1919 transcribed RNA sequence OS=Wollemia nobilis OX=56998 PE=3 SV=1;tr|A0A0D6QZJ0|A0A0D6QZJ0_ARACU Uncharacterized protein OS=Araucaria cunninghamii OX=56994 PE=3 SV=1

tr|B8LRY7|B8LRY7_PICSI Uncharacterized protein OS=Picea sitchensis OX=3332 PE=2 SV=1

tr|B8LRY5|B8LRY5_PICSI Uncharacterized protein OS=Picea sitchensis OX=3332 PE=2 SV=1

tr|B8LM26|B8LM26_PICSI Uncharacterized protein OS=Picea sitchensis OX=3332 PE=2 SV=1

tr|A9NWA5|A9NWA5_PICSI Uncharacterized protein OS=Picea sitchensis OX=3332 PE=2 SV=1

tr|A9NV59|A9NV59_PICSI Uncharacterized protein OS=Picea sitchensis OX=3332 PE=2 SV=1

tr|A9NPE8|A9NPE8_PICSI Uncharacterized protein OS=Picea sitchensis OX=3332 PE=2 SV=1

tr|A0A0D6QXP6|A0A0D6QXP6_ARACU Uncharacterized protein OS=Araucaria cunninghamii OX=56994 PE=3 SV=1

tr|A0A0D6QR30|A0A0D6QR30_ARACU Uncharacterized protein OS=Araucaria cunninghamii OX=56994 PE=3 SV=1;tr|A0A0C9QSE0|A0A0C9QSE0_9SPER TSA: Wollemia nobilis Ref_Wollemi_Transcript_11965_1333 transcribed RNA sequence OS=Wollemia nobilis OX=56998 PE=3 SV=1

tr|A0A0D6R6U3|A0A0D6R6U3_ARACU Uncharacterized protein OS=Araucaria cunninghamii OX=56994 PE=3 SV=1;tr|A0A0C9SAT1|A0A0C9SAT1_9SPER TSA: Wollemia nobilis Ref_Wollemi_Transcript_2025_2303 transcribed RNA sequence OS=Wollemia nobilis OX=56998 PE=3 SV=1

tr|A0A0D6QRL3|A0A0D6QRL3_ARACU Uncharacterized protein OS=Araucaria cunninghamii OX=56994 PE=4 SV=1;tr|A0A0C9QM40|A0A0C9QM40_9SPER TSA: Wollemia nobilis Ref_Wollemi_Transcript_25193_3139 transcribed RNA sequence OS=Wollemia nobilis OX=56998 PE=4 SV=1

tr|A0A0D6R9M0|A0A0D6R9M0_ARACU Uncharacterized protein OS=Araucaria cunninghamii OX=56994 PE=4 SV=1;tr|A0A0C9RRM8|A0A0C9RRM8_9SPER TSA: Wollemia nobilis Ref_Wollemi_Transcript_18828_1885 transcribed RNA sequence OS=Wollemia nobilis OX=56998 PE=4 SV=1;tr|C9

tr|A0A0D6R407|A0A0D6R407_ARACU Uncharacterized protein OS=Araucaria cunninghamii OX=56994 PE=4 SV=1;tr|A0A0C9S1C0|A0A0C9S1C0_9SPER TSA: Wollemia nobilis Ref_Wollemi_Transcript_25761_1196 transcribed RNA sequence OS=Wollemia nobilis OX=56998 PE=4 SV=1;tr|B8

tr|A0A0D6QT13|A0A0D6QT13_ARACU Uncharacterized protein OS=Araucaria cunninghamii OX=56994 PE=4 SV=1;tr|A9NPT3|A9NPT3_PICSI Uncharacterized protein OS=Picea sitchensis OX=3332 PE=2 SV=1;tr|A0A0D6R740|A0A0D6R740_ARACU Uncharacterized protein OS=Araucaria cun

tr|D5AA85|D5AA85_PICSI Uncharacterized protein OS=Picea sitchensis OX=3332 PE=2 SV=1;tr|A0A0C9S5D2|A0A0C9S5D2_9SPER TSA: Wollemia nobilis Ref_Wollemi_Transcript_19711_1759 transcribed RNA sequence OS=Wollemia nobilis OX=56998 PE=4 SV=1

tr|A9NPE7|A9NPE7_PICSI Uncharacterized protein OS=Picea sitchensis OX=3332 PE=2 SV=1;tr|A0A0D6QSB4|A0A0D6QSB4_ARACU Uncharacterized protein OS=Araucaria cunninghamii OX=56994 PE=4 SV=1;tr|A0A0C9S3Z1|A0A0C9S3Z1_9SPER TSA: Wollemia nobilis Ref_Wollemi_Transc

tr|A9NT20|A9NT20_PICSI Uncharacterized protein OS=Picea sitchensis OX=3332 PE=2 SV=1;tr|A0A3G6J9L9|A0A3G6J9L9_PINTA Chitinase-like protein 1 OS=Pinus taeda OX=3352 GN=GH19a PE=2 SV=1

tr|A9P0U3|A9P0U3_PICSI Uncharacterized protein OS=Picea sitchensis OX=3332 PE=2 SV=1;tr|A9NX36|A9NX36_PICSI Uncharacterized protein OS=Picea sitchensis OX=3332 PE=2 SV=1

tr|B8LQ09|B8LQ09_PICSI Uncharacterized protein OS=Picea sitchensis OX=3332 PE=2 SV=1;tr|A9NZQ3|A9NZQ3_PICSI Uncharacterized protein OS=Picea sitchensis OX=3332 PE=2 SV=1

tr|D5A7T4|D5A7T4_PICSI Uncharacterized protein OS=Picea sitchensis OX=3332 PE=2 SV=1;tr|B8LPY2|B8LPY2_PICSI Uncharacterized protein OS=Picea sitchensis OX=3332 PE=2 SV=1;tr|A0A0D6QTC1|A0A0D6QTC1_ARACU Uncharacterized protein OS=Araucaria cunninghamii OX=56

tr|B8LQR0|B8LQR0_PICSI Uncharacterized protein OS=Picea sitchensis OX=3332 PE=2 SV=1;tr|C0PQ35|C0PQ35_PICSI Uncharacterized protein OS=Picea sitchensis OX=3332 PE=2 SV=1;tr|B8LL07|B8LL07_PICSI Uncharacterized protein OS=Picea sitchensis OX=3332 PE=2 SV=1;t

tr|C0PSY5|C0PSY5_PICSI Uncharacterized protein OS=Picea sitchensis OX=3332 PE=2 SV=1;tr|C0PR00|C0PR00_PICSI Uncharacterized protein OS=Picea sitchensis OX=3332 PE=2 SV=1;tr|A9NUR3|A9NUR3_PICSI Uncharacterized protein OS=Picea sitchensis OX=3332 PE=2 SV=1;t

tr|A9NVN4|A9NVN4_PICSI Uncharacterized protein OS=Picea sitchensis OX=3332 PE=2 SV=1;tr|Q8RVJ2|Q8RVJ2_PINPS Putative glucosidase-like protein (Fragment) OS=Pinus pinaster OX=71647 PE=2 SV=1;tr|A0A075M683|A0A075M683_9SPER Putative glucan-endo-1,3-beta-gluco

tr|A9P2N8|A9P2N8_PICSI Xyloglucan endotransglucosylase/hydrolase OS=Picea sitchensis OX=3332 PE=2 SV=1;tr|Q588B9|Q588B9_CRYJA Xyloglucan endotransglucosylase/hydrolase OS=Cryptomeria japonica OX=3369 PE=2 SV=1;tr|A9NZ22|A9NZ22_PICSI Xyloglucan endotransglu

Table 3 Sugar concentrations (conc. mg/µl) of megagametophyte fluids and archegonial chamber fluids from four individuals of *Cycas revoluta*

	megagametophtye fluid											
sample	22.1	22.2	22.3	26.1	26.2	26.3	34.1	34.2	34.3	35.1	35.2	35.3
glucose	20.4	17.5	19.4	18.5	19.9	19.9	16.6	17.4	16.8	33.5	35.6	35.4
glucose %	81.6	79.9	79.5	91.6	91.7	92.1	94.3	94.6	94.4	89.6	90.4	90.1
fructose	3.0	2.5	2.9	1.7	1.8	1.7	1	1	1	3.9	3.8	3.9
fructose %	12.0	11.4	11.9	8.4	8.3	7.9	5.7	5.4	5.6	10.4	9.6	9.9
sucrose	1.6	1.9	2.1	-	-	-	-	-	-	-	-	-
sucrose%	6.4	8.7	8.6	-	-	-	-	-	-	-	-	-
total conc.	25	21.9	24.4	20.2	21.7	21.6	17.6	18.4	17.8	37.4	39.4	39.3
pectins*	15.5	15	16.9	9.1	10.8	10.8	7.2	8.0	7.5	21	26.2	26.1

	archegonial chamber fluid											
sample	19.1	19.2	19.3	29.1	29.2	29.3	38.1	38.2	38.3	40.1	40.2	40.3
glucose	3.2	3.1	2.7	19.4	20.1	20.4	15.3	14.1	15.3	16	16.2	15.5
glucose %	52.5	47.0	45.8	91.5	91.4	91.5	79.3	77.5	78.5	90.4	91.0	90.6
fructose	1.1	1.1	1.1	1.8	1.9	1.9	2.5	2.7	2.8	1.7	1.6	1.6
fructose %	18.0	16.7	18.6	8.5	8.6	8.5	13.0	14.8	14.4	9.6	9.0	9.4
sucrose	1.8	2.4	2.1	-	-	-	1.5	1.4	1.4	-	-	-
sucrose%	29.5	36.4	35.6	-	-	-	7.8	7.7	7.2	-	-	-
total conc.	6.1	6.6	5.9	21.2	22	22.3	19.3	18.2	19.5	17.7	17.8	17.1
pectins*	2.7	2.8	2.2	4.7	5	5	7.3	6.8	7.2	7.3	7.3	7.2

Dashes indicate not detected. Sample numbers indicate the technical replicate based on the number following the decimal (i.e. 19.2, is the second replicate for sample 19). *Pectins were quantified using Galacturonic acid equivalent concentration

Table 4 Amino acid concentrations (pmol/µl) of megagametophyte fluids and archegonial chamber fluids from four individuals of *Cycas revoluta*

	megagametophyte fluid											
sample	22.1	22.2	22.3	26.1	26.2	26.3	34.1	34.2	34.3	35.1	35.2	35.3
ALA	595.9	586.6	598.1	-		-	-	-	-	68.7	68	64.7
ARG	-	-	-	-	-	-	-	-	-	-	-	-
ASP	141.7	166.7	167.8	-	-	-	-	-	-	-	-	-
GLU	1376.2	1368.2	1350.1	-	-	-	-	-	-	-	-	-
GLY	233.9	200.4	222.3	122.3	134.9	146	106.9	137	123.4	107	112.9	114.4
HYS	-	-	-	-	-	-	-	-	-	61.8	63.5	64.2
ILE	-	-	-	114.2	135.9	128	105.2	119.1	119.2	145.5	147.6	140.6
LEU	24.6	24.2	23.7	-	-	-	-	-	-	-	-	-
LYS	-	-	-	-	-	-	-	-	-	-	-	-
MET	25	27	26.1	-	-	-	-	-	-	-	-	-
PHE	41.2	40.7	41.4	-	-	-	-	-	-	15.7	15.6	12.9
PRO	730.1	679.3	684.3	-	-	-	-	-	-	-	-	-
SER	50.6	50.2	50	-	-	-	-	-	-	-	-	-
THR	76	65.1	67	-	-	-	-	-	-	-	-	-
TYR	58	52.4	52.9	-	-	-	-	-	-	-	-	-
VAL	87.5	58.8	60.3	-	-	-	-	-	-	-	-	-
AABA	-	-	-	-	-	-	-	-	-	-	-	-
BALA	43.7	40.5	38.5	-	-	-	-	-	-	28.2	30.2	26.7
ORN	111.1	106.9	107.4	43.3	47.9	48.1	39	45	43.4	75.3	79.8	49.5
total	3595.5	3467	3489.9	279.8	318.7	322.1	251.1	301.1	286	502.2	517.6	473.0

	archegonial chamber fluid											
sample	19.1	19.2	19.3	29.1	29.2	29.3	38.1	38.2	38.3	40.1	40.2	40.3
ALA	77.9	76.6	79.5	192.5	194.0	186.7	2327.7	2375.0	2428.0	840.0	845.6	850.0
ARG	-	-	-	-	-	-	123.6	95.7	94.7	-	-	-
ASP	-	-	-	113.1	115.0	114.8	402.2	417.9	423.0	79.5	81.0	124.8
GLU	1099.1	1131.4	1171.8	161.3	163.2	162.8	4988.0	4940.2	5059.3	4449.5	4457.8	4456.4
GLY	94.8	97.8	102.3	308.6	307.6	437.6	692.6	661.0	673.8	423.0	428.8	426.7
HYS	-	-	-	-	-	-	128.3	106.3	109.5	-	-	-
ILE	-	-	-	-	-	-	141.3	143.5	145.4	-	-	-
LEU	16.3	17.2	17.8	74.3	74.2	73.6	213.8	215.5	223.6	33.4	33.0	32.8
LYS	-	-	-	56.7	51.3	49.8	116.8	117.9	115.2	-	-	-
MET	48.5	49.8	51.3	-	-	-	97.5	93.8	95.6	40.4	44.6	41.0
PHE	13.7	14.0	15.4	38.7	38.1	36.0	165.3	169.6	173.2	40.5	41.6	41.7
PRO	157.0	165.5	166.1	-	-	-	1322.8	1332.0	1353.7	945.0	950.4	953.0
SER	207.7	213.3	220.7	52.1	56.0	51.3	-	-	-	-	-	-
THR	36.1	33.2	34.6	-	-	-	172.5	163.7	166.9	-	-	-
TYR	-	-	-	42.8	42.7	38.0	201.7	192.0	193.7	61.7	65.7	64.4
VAL	29.5	30.3	31.5	82.2	84.0	86.4	280.0	283.7	287.2	336.2	335.5	339.0
AABA	-	-	-	-	-	-	61.6	58.9	58.0	84.1	86.6	84.6
BALA	-	-	-	86.7	90.8	86.4	218.3	210.0	212.5	204.1	218.0	218.0
ORN	41.6	45.2	46.2	-	-	-	-	-	-	-	-	-
total	1822.2	1874.3	1937.2	1209	1216.9	1323.4	11654	11576.7	11813.3	7537.4	7588.6	7632.4

Dashes indicate not detected. Several amino acids were not found in any sample: Asparagine, Cystine, Glutamine, β amino butyric acid, γ amino butyric acid, Citrulline, Hydroxyproline, and Taurine. Sample numbers indicate the technical replicate based on the number following the decimal (i.e. 19.2, is the second replicate for sample 19)
