# Supplementary material for: Composition of Sexual Fluids in Cycas revoluta Ovules During Pollination and Fertilization
Source: Bot Rev. 2022 Jan 1;88(4):453–84. doi: 10.1007/s12229-021-09271-1 (PMC9726676; doi:10.1007/s12229-021-09271-1)
Supplement: Supplementary file 2 — Supplementary file2 (DOCX 25 kb) Methods S1 Analytical methods used for proteomics, as well as carbohydrate and amino acid analysis. [file 12229_2021_9271_MOESM2_ESM.docx]

**Supporting Information Methods**

**Proteomics of MF and ACF**

Basic Local Alignment Search Tool for proteins (BLASTp) was used to annotate the predicted proteins identified by mass spectrometry. The FASTA files generated from Scaffold Software containing the amino acid sequences, and all hits for each LC–MS/MS run were blast searched against the Universal Protein Resource (Uniprot) database accessed online (<https://www.uniprot.org/blast/>). The BLAST hits were filtered at a threshold E-value<e−5. Descriptive names, gene ontology (GO), and annotations were collected (Silva et al. 2020).

Scaffold Software (v 4.6.0 Proteome Software Inc., Portland, OR, USA) was used to compare the sexual fluid proteome through stages MF and ACF. A threshold of a two peptide per protein minimum was used. Peptides had to exceed a 95 % threshold, and have a FDR of less than 1 %. The mass spectrometry proteomics data have been deposited to the ProteomeXchange Consortium via the PRIDE partner repository with the dataset identifier PXD029259 and 10.6019/PXD029259.

**Label-free quantitation mass spectrometry of MF and ACF**

For the Spearman analysis, the Perseus software platform was used to analyze the set of 22 identified proteins common to both MF and ACF. The protein distributions were normalized before creating Spearman scatterplots. ANOVA tests of individual plant differences of ACF and then, separately, of MF showed no significant differences (not shown). Comparisons of protein expression were corrected using Benjamini-Hochberg at FDR 0.02. Principal Component Analysis of biological groupings showed no pattern: proteins neither clustered by individual nor by fluid type (not shown).

**Proteomics of Pollination Drops**

Ammonium bicarbonate (50µL of 25 mM) was added to each sample. The samples were reduced with 2.5µL of 200mM DTT (45 min at 37 °C), and cysteine sulfhydryls were alkylated with 5µL of 200mM iodoacetamide (45 min at 37 °C in darkness), and quenched with 5µL, 200mM DTT. One microgram of trypsin (Promega) was added to each sample and digested at 37 °C for 16 hr. The samples were pooled following digestion. The pooled sample was de-salted on a Waters HLB Oasis column, speed vac-concentrated and then stored at -80 °C before LC-MS/MS analysis.

Thermo Scientific C18 Stage Tips SP301 (200 µL) were used to desalt peptides. Following binding and washing, peptides were eluted with 80 µL (80 % v/v acetonitrile, 0.1 % v/v formic acid), speed vac-concentrated to near dryness and rehydrated with 2 % acetonitrile, 0.1 % formic acid, water. After rehydration with 20 µL 2 % acetonitrile, 0.1 % formic acid, water a 5-microlitre injection was separated by on-line reverse phase chromatography using a Thermo Scientific EASY-nLC 1000 system with a reverse-phase pre-column Magic C18-AQ (100 µm I.D., 2.5 cm length, 5 µm, 100 Å, and an in-house prepared reverse phase nano-analytical column Magic C-18AQ (75 µm I.D., 15 cm length, 5 µm, 100 Å, Michrom BioResources Inc, Auburn, CA), at a flow rate of 300 nl min^-1^. The chromatography system was coupled on-line with an Orbitrap Fusion Tribrid mass spectrometer (Thermo Fisher Scientific, San Jose, CA) equipped with a Nanospray Flex NG source (Thermo Fisher Scientific). Solvents were A: 2 % acetonitrile, 0.1 % formic acid; B: 90 % acetonitrile, 0.1 % formic acid. Samples were separated by a 90 min gradient (0 min: 3 % B; 75 min: 30 % B; 10 min: 45 % B; 5 min: 40 % B; 5 min: 100 % B; hold 5min: 100 % B).

The Orbitrap Fusion Tune 3.0 software instrument parameters were as follows for Orbitrap iontrap with high energy collision dissociation (HCD) and CID fragmentation: Nano-electrospray ion source with spray voltage 2.55 kV, capillary temperature 275 ℃. Survey MS1 scan m/z range 400-2000 profile mode, resolution 120,000 full width at half maximum (FWHM) at 200 m z^-1^ one microscan with maximum inject time 50 ms. The Siloxane mass 445.120024 was used as lock mass for internal calibration. Data-dependent acquisition Orbitrap survey spectra were scheduled at least every 3 s, with the software determining ‘Top-speed’ number of MS/MS acquisitions during this period. The automatic gain control (AGC) target values for Fourier transform mass spectrometry (FTMS) and multi stage mass spectrometry (MS^n^) were 400,000 and 5,000 respectively. The most intense ions charge state 2-5 exceeding 20,000 counts were selected for HCD or CID ion trap MS/MS fragmentation with detection in centroid mode. Monoisotopic Precursor Selection (MIPS) was enabled and dynamic exclusion settings were as follows: repeat count, 2; repeat duration, 10 s; exclusion duration, 10 s with a 10 ppm mass window. The ddMS2 ion trap high energy collision dissociation (IT HCD) scan used a quadrupole isolation window of 1.6 Da; IonTrap rapid scan rate centroid detection first mass 100 m z^-1^, 1 microscan, 250 ms maximum injection time and stepped collision energy 33 % ± 3. The ddMS2 IT CID scan used a quadrupole isolation window of 1.6 Da; IonTrap rapid scan rate centroid detection auto normal scan range, activation time 10 ms 1 microscan, 250 ms maximum injection time and a fixed collision energy 35 %. Known keratin and trypsin autolysis masses were excluded from acquisition for the entire LC gradient.

Raw files were created by XCalibur 4.1.31.9 (Thermo Scientific) software and analysed with Proteome Discoverer 2.2.0388 software suite (Thermo Scientific). Parameters for the Spectrum Selection to generate peak lists of the HCD spectra (activation type: HCD; signal to noise ratio (s/n) cut-off: 1.5; total intensity threshold: 0; minimum peak count: 5; precursor mass: 350-5000 Da) The peak lists were submitted to an in-house Mascot 2.5.1 server Uniprot-Swissprot 20180404 (557,012 sequences; 199,714,119 residues) and UniprotKB 20161219 (71,594,789 sequences; 24,046,658,667 residues) database search as follows: precursor tolerance 8 ppm; MS/MS tolerance 0.6 Da; trypsin enzyme 1 missed cleavages; FT-ICR instrument type; fixed modification: carbamidomethyl (C); variable modifications: oxidation (M) and deamidated (N,Q). Percolator settings were as follows: Max delta Cn 0.05; Target False Detection Rate (FDR) strict 0.01, Target FDR relaxed 0.05 with validation based on q-Value.

The identified proteins were loaded, validated, and filtered using Scaffold Software (v 4.0 Proteome Software, Portland, Inc., Portland, OR, USA). The parameters used to accept an identification were as follows: peptide spectrum match; FDRs, peptides, and protein levels. All results were filtered with FDR of ≤ 1%, peptide threshold of 95% and a minimum of two peptides per protein identification. The mass spectrometry proteomics data have been deposited to the ProteomeXchange Consortium via the PRIDE partner repository with the dataset identifier PXD029231 and 10.6019/PXD029231.

**Sugar Analysis of MF and ACF**

Samples were analyzed for sugar content using isocratic high-performance liquid chromatography (HPLC). The sample and standard solutions containing glucose, fructose, sucrose, and glucuronic acid (20 µl) were injected into a Waters 600 E pump system. The mobile phase was deionized water. The flow rate was set at 0.5 ml min^-1^ and column temperature at 90 °C. Sugars were separated in a Waters Sugar-Pak I (6.5–300 mm) column and identified with a Waters 2410 refractive index detector. The concentration of each single sugar was calculated by comparing the area under the chromatogram peaks with standards using the software Clarity (DataApex). In addition, the concentration of pectins was measured. It was expressed as galacturonic acid equivalents (mg ml^-1^). These were run identically to previously published sugars analyzed in pollination drops (Nepi *et al*., 2017), which allowed comparison between data generated with the cycad work and the previous pollination drop data.

**Amino Acid Analysis of MF and ACF**

Amino acid analysis was performed by gradient HPLC with an AccQtag system column (15 mm x 4.6 mm) maintained at 37 °C and a Waters 470 scanning fluorescence detector (excitation at 295 nm, detection at 350 nm). An AccQtag system buffer and a 6:4 acetonitrile-water solution were used in gradient as mobile phase at a flow rate of 1.0 ml min^-1^. The selected volume of each reconstituted sample was amino acid derivatized (Cohen & Micheaud, 1993) with AQC fluorescent reagent and 0.02 M borate buffer (pH 8.6), according to AccQtag protocol (Waters Corp.). In addition to 19 of 20 protein-associated amino acids (tryptophan is not detectable with this method), standards for 9 non-protein amino acids (β-alanine, citrulline, α-aminobutyric acid (AABA), β-aminobutyric acid (BABA), γ-aminobutyric acid (GABA), hydroxyproline, ornithine and taurine) were also used. The concentration of each individual amino acid was calculated by comparing the area under the chromatogram peaks with standards using the software Clarity (DataApex). These were run identically to previously published sugars analyzed in pollination drops (Nepi *et al*., 2017), which allowed comparison with that data.
