# Supplementary material for: Composition of Sexual Fluids in Cycas revoluta Ovules During Pollination and Fertilization
Source: Bot Rev. 2022 Jan 1;88(4):453–84. doi: 10.1007/s12229-021-09271-1 (PMC9726676; doi:10.1007/s12229-021-09271-1)
Supplement: Supplementary file 3 — Supplementary file3 (PDF 58 kb) Fig. S1 Non-metric multidimensional scaling plot of amino acid and carbohydrate composition. [file 12229_2021_9271_MOESM3_ESM.pdf]

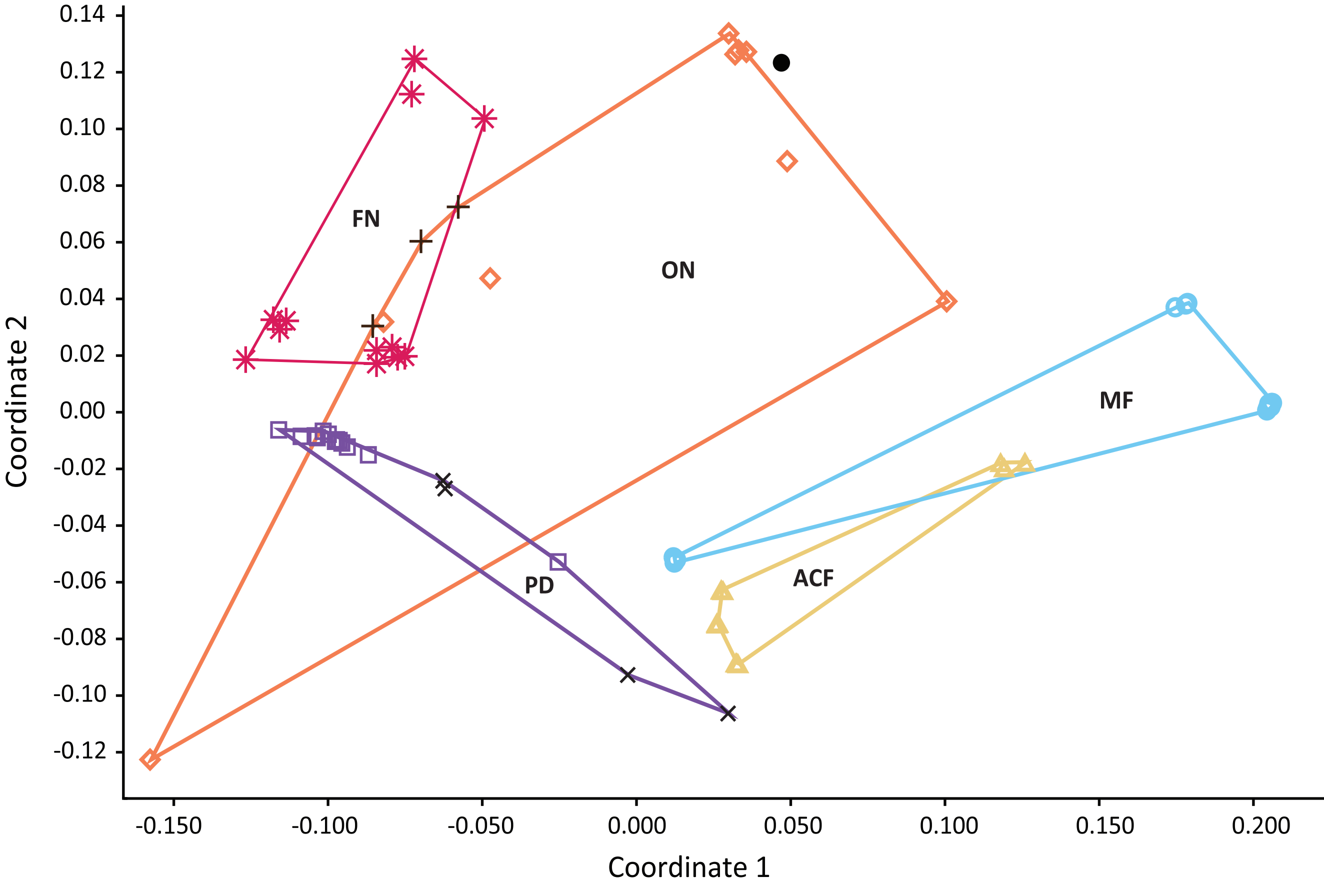

0.14
0.12
0.10
0.08
0.06
0.04
0.02
0.00
-0.02
-0.04
-0.06
-0.08
-0.10
-0.12
Coordinate 2
-0.150
-0.100
-0.050
0.000
0.050
0.100
0.150
0.200
Coordinate 1
FN
ON
MF
ACF
PD

Fig. S1 Non-metric multidimensional scaling (NMDS) plot of amino acid and carbohydrate composition of megagametophyte (MF, blue) and archegonial chamber fluid ACF, yellow) compared to pollination drop of a cycad (black dot), pollination drops of Gnetales and *Ginkgo* (ON, orange), conifers and Pinaceae (PD, purple), and floral nectars of angiosperms (FN, magenta). Megagametophyte and archegonial chamber fluids plot to the right of the plot, mainly based on low carbohydrate concentrations of mainly glucose. MF and ACF are high in amino acid concentrations (lower part of plot). Data for angiosperm nectar and gymnosperm pollination drops are from Nepi et al. (2017)
